# Astrocyte‐Associated ABCA1/APOE Responses Accompany Hippocampal Lipid Remodeling and Ectopic Neuronal Accumulation During Epileptogenesis

**DOI:** 10.1002/advs.78329

**Published:** 2026-10-18

**Authors:** Lu Chen, Xiao‐Rui Shi, Jia‐Yi Chen, Ying‐Ting Wang, Le Du, Yang Ni, Jun‐Min Chen, An‐Chen Guo, Qian Wu, Jian‐Ping Wu, Qun Wang

**Affiliations:** ^1^ Department of Neurology Beijing Tiantan Hospital Capital Medical University Beijing China; ^2^ China National Clinical Research Center For Neurological Diseases Beijing Tiantan Hospital Capital Medical University Beijing China; ^3^ National‐Local Joint Engineering Research Center For Drug Research and Development (R&D) of Neurodegenerative Diseases Dalian Medical University Dalian China; ^4^ School of Chemistry Chemical Engineering and Life Sciences Wuhan University of Technology Wuhan China; ^5^ Department of Neurology First Affiliated Hospital Kunming Medical University Kunming China; ^6^ Beijing Key Laboratory of Drug and Device Research and Development for Cerebrovascular Diseases Beijing China; ^7^ Department of Pharmacology Hubei University of Medicine Shiyan China; ^8^ Department of Neurology First Affiliated Hospital of Zhengzhou University Zhengzhou China

**Keywords:** ABCA1, astrocytes, ectopic neuronal accumulation, epileptogenesis, lipid metabolism, temporal lobe epilepsy

## Abstract

Temporal lobe epilepsy (TLE) is characterized by progressive hippocampal remodeling, including reactive astrogliosis and aberrant neuronal positioning. Although lipid disturbances are implicated in epileptogenesis, how astrocyte‐associated lipid responses relate to hippocampal remodeling remains poorly understood. Here, human TLE and kainic acid (KA)‐treated mouse transcriptomic datasets identified ATP‐binding cassette transporter A1 (ABCA1)/Abca1 as a nominal astrocyte‐associated candidate, while KA‐ and pilocarpine‐induced status epilepticus (SE) elicited astrocyte‐associated ABCA1/apolipoprotein E (APOE) responses. Ectopic granule cells and immature neurons accumulated in the hilus and were spatially associated with ABCA1‐positive cells, including astrocytic profiles; perilipin 2 (PLIN2)‐positive puncta were nearby. Pretreatment with the retinoid X receptor (RXR) agonist bexarotene increased hippocampal ABCA1 expression, delayed severe KA‐induced behavioral seizure onset, and was associated with fewer chronic spontaneous seizures and reduced ectopic neuronal accumulation. Post‐SE bexarotene administration was associated with lower seizure frequency, ectopic neuronal accumulation, and astrocytic marker‐positive cell densities. Multi‐omic profiling identified bexarotene‐associated metabolic and lipidomic changes; transmission electron microscopy (TEM) showed a smaller mean hilar neuronal lipid‐droplet area per image, and protein analyses identified early changes in p38 and extracellular signal‐regulated kinase 1/2 (Erk1/2) phosphorylation. Together, these findings warrant further investigation of astrocyte‐associated lipid pathways as potential therapeutic targets in epilepsy‐associated hippocampal remodeling.

## Graphical abstract

During kainic acid‐induced epileptogenesis, astrocyte‐associated ATP‐binding cassette transporter A1 (ABCA1) and apolipoprotein E (APOE) responses accompany ectopic neurons and exploratory lipid alterations. In separate cohorts, bexarotene pretreatment and treatment on days 14–21 are associated with fewer ectopic hilar neurons and lower chronic seizure frequency; treatment on days 7–14 is associated with higher ABCA1 and smaller mean hilar neuronal lipid‐droplet area.

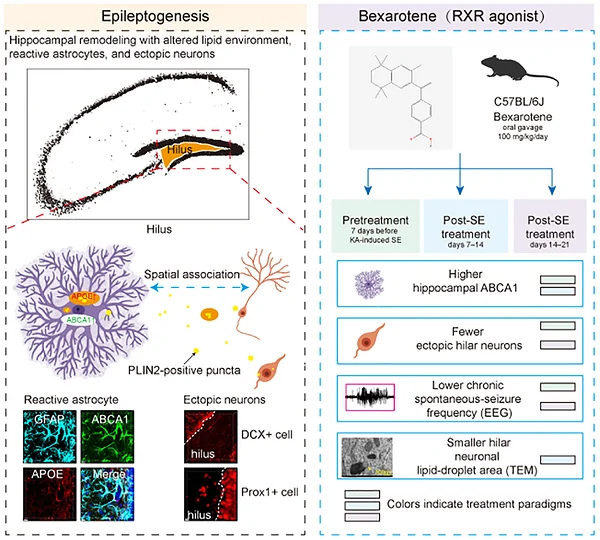

**Abbreviations**
ABCA1: ATP‐binding cassette transporter A1
APOE: Apolipoprotein E
Cer: Ceramides
EEG: Electroencephalography
GEO: Gene expression omnibus
KA: Kainic acid
LARAs: Lipid‐accumulated reactive astrocytes
LD: Lipid droplet
MSI: Mass spectrometry imaging
NCM: Neuronal conditioned medium
PC: Phosphatidylcholine
PCA: Principal component analysis
PE: Phosphatidylethanolamine
POS: Positive ion mode
SE: Status epilepticus
SGZ: Subgranular zone
SRS: Spontaneous recurrent seizures
TEM: Transmission electron microscopy
TG: Triglyceride
TLE: Temporal lobe epilepsy

## 1 Introduction

Temporal lobe epilepsy (TLE) is one of the most common and pharmacoresistant neurological disorders, characterized by progressive hippocampal remodeling, neuroinflammation, aberrant neurogenesis, and spontaneous recurrent seizures (SRS) [1, 2, 3]. Although current anti‐seizure medications (ASMs) effectively suppress neuronal hyperexcitability, they do not prevent pathological network reorganization, and approximately 30%–40% of patients remain drug‐resistant [4]. Consequently, increasing attention has shifted from seizure suppression toward identification of disease‐modifying mechanisms that regulate pathological network remodeling during epileptogenesis.

Beyond classical cytokine‐mediated signaling pathways, emerging evidence suggests that metabolic stress, particularly lipid dysregulation, represents a critical but underexplored driver of neuroinflammatory responses in epilepsy [5, 6]. The brain is highly enriched in lipids that are essential for energy metabolism, membrane homeostasis, and neuroinflammatory signaling [7]. Lipidomic analyses of the epileptic hippocampus have revealed extensive remodeling of structural phospholipids, including phosphatidylcholine (PC) and phosphatidylethanolamine (PE), accompanied by abnormal accumulation of triacylglycerols and cholesteryl esters [8, 9]. These alterations are increasingly recognized not merely as passive metabolic consequences of seizures, but as active contributors to epileptogenic remodeling. Excessive lipid dysregulation promotes lipid droplet (LD) accumulation and lipotoxic stress, thereby contributing to a lipid‐remodeled hippocampal microenvironment accompanied by glial activation and inflammatory changes [10, 11, 12]. Notably, lipid‐accumulated reactive astrocytes (LARAs) have recently been identified as a distinct pathological population in epilepsy where excessive neuron‐to‐astrocyte lipid transfer reshapes astrocytic phenotypes and contributes to disease progression [13]. However, how seizure‐associated lipid alterations relate to hippocampal structural remodeling and how astrocytes respond to the altered lipid microenvironment remain incompletely understood.

During epileptogenesis, status epilepticus (SE) induces massive neuronal activity and injury, generating oxidized membrane lipids and other metabolic products [14, 15]. Because neurons possess limited capacity for lipid storage and oxidation, they rely heavily on astrocytes to buffer and detoxify excessive lipids. Astrocytes therefore function as essential regulators of local metabolic and inflammatory homeostasis through lipid uptake and clearance mechanisms that resemble non‐canonical efferocytosis of neuronal debris [12, 16]. Importantly, these lipid‐handling processes occur in parallel with another hallmark feature of epileptogenesis ─ aberrant neurogenesis and ectopic neuronal migration within the hippocampus [17]. Following seizures, neural progenitors in the subgranular zone (SGZ) become hyperactivated and migrate ectopically into the hilus and molecular layer, where they integrate pathological circuits and contribute to spontaneous seizure generation [18, 19, 20]. Intriguingly, signaling pathways regulating neuronal migration, including Reelin pathway and its receptors ApoER2 and VLDLR are closely linked to lipid transport systems mediated by apolipoprotein E (APOE) [21, 22]. We previously demonstrated that Disrupted‐in‐Schizophrenia 1 (DISC1) promotes ectopic migration of newborn neurons following epilepsy via the Reelin‐ApoER signaling axis and observed that reactive astrocytes actively “encircle” these DISC1^+^ migrating neurons [23, 24]. Given the central role of astrocytes in APOE‐dependent lipid transport and metabolic support within the injured brain [25], these findings raise the possibility that lipid dysregulation and ectopic neuronal migration may be spatially and functionally associated through local neuron–astrocyte interactions. However, whether seizure‐associated astrocytic lipid‐handling responses occur in spatial association with ectopically located neurons, and whether these processes are linked to hippocampal remodeling during epileptogenesis, remains unknown.

ATP‐binding cassette transporter A1 (ABCA1) is a key regulator of cellular lipid efflux that mediates the transfer of cholesterol and phospholipids to apolipoproteins, such as APOE, thereby supporting lipoprotein particle formation and lipid redistribution within the central nervous system [26]. In astrocytes, ABCA1 contributes to lipid homeostasis and may influence inflammatory and tissue‐repair responses following neurological injury [27]. Consistent with this role, multiple lines of evidence from human TLE studies have begun to reveal disrupted lipid handling in the epileptic hippocampus. Mass spectrometry imaging (MSI) of hippocampal tissues from TLE patients has demonstrated widespread alterations in phospholipid metabolism [8]. Complementing these lipidomic findings, bioinformatic analyses of transcriptomic datasets have further identified significantly increased ABCA1 expression in reactive astrocytes in human TLE and rat epilepsy models [28], suggesting an astrocyte‐associated ABCA1 response during epileptogenesis. Moreover, recent studies have described a distinct population of LARAs, a pathological astrocyte subset whose emergence is closely associated with dysregulated neuron‐to‐astrocyte lipid transfer and disease progression [13]. Despite these observations, the relationship between astrocytic ABCA1‐associated responses, ectopically located neuronal populations, and hippocampal remodeling during epileptogenesis remain unclear.

Here, we hypothesized that seizure‐induced lipid alterations are associated with a hippocampal microenvironment showing reactive astrocytic responses and aberrant neuronal positioning. To investigate this hypothesis, we combined untargeted lipidomics, metabolomics, ultrastructural analysis, transcriptomic data mining, and in vivo electrophysiological monitoring in a kainic acid (KA)‐induced epilepsy model, with ABCA1 and APOE expression further examined in a parallel pilocarpine‐induced epilepsy model. Using pharmacological activation of RXR signaling with bexarotene, we further investigated whether treatment was associated with changes in seizure‐related outcomes and hippocampal cellular remodeling. Our findings identify exploratory seizure‐associated hippocampal lipidomic differences and astrocyte‐associated ABCA1/APOE responses that occur alongside ectopic neuronal accumulation and glial remodeling. Furthermore, bexarotene administration was associated with metabolic and molecular remodeling, delayed onset of severe behavioral seizures after pretreatment, lower chronic spontaneous seizure frequency in the monitored cohorts, and lower ectopic neuronal accumulation, supporting further investigation of astrocyte‐associated lipid pathways as potential therapeutic targets in TLE.

## 2 Results

### 2.1 Hippocampal Lipid Profiles and Lipid Droplet Measures after KA‐Induced SE

To determine whether epileptogenesis is associated with large‐scale metabolic remodeling within the hippocampus, we first performed untargeted lipidomic profiling in hippocampal tissues collected 14 days after KA‐induced SE. Because most candidate structural phospholipids and neutral lipids were detected in positive ion mode (POS), subsequent multivariate and pathway analyses were performed using the POS dataset. The partial least squares‐discriminant analysis (PLS‐DA) score plot showed separation between the KA and Con groups in the hippocampal lipidomic dataset (Figure 1A). Using the prespecified nominal screening criteria based on *p* value, VIP score, and fold change, volcano plot analysis identified 67 candidate lipid species (16 increased and 51 decreased) between the two groups (Figure 1B). However, no individual lipid species remained significant after Benjamini–Hochberg correction (minimum *q* = 0.567); these changes were therefore considered exploratory. Heatmap and subclass annotation showed distinct abundance patterns dominated by PC, PE, and TG species (Figure 1C,D). Multiple PC and PE species were decreased in the KA group, whereas TG species showed heterogeneous, although predominantly decreased, changes. These exploratory findings suggest coordinated remodeling of hippocampal lipid composition rather than a uniform alteration of a single lipid class. Direction‐specific KEGG analysis identified no false discovery rate (FDR)‐significant pathway among increased lipids (minimum q = 0.609). Exploratory analysis of decreased lipids highlighted lipid‐associated pathways, including cholesterol metabolism, glycerolipid metabolism, fat digestion and absorption, and insulin resistance (Figure 1E). However, no pathway remained FDR significant after correction (minimum *q = 0.145*); these pathway‐level observations were likewise considered exploratory.

**FIGURE 1:**
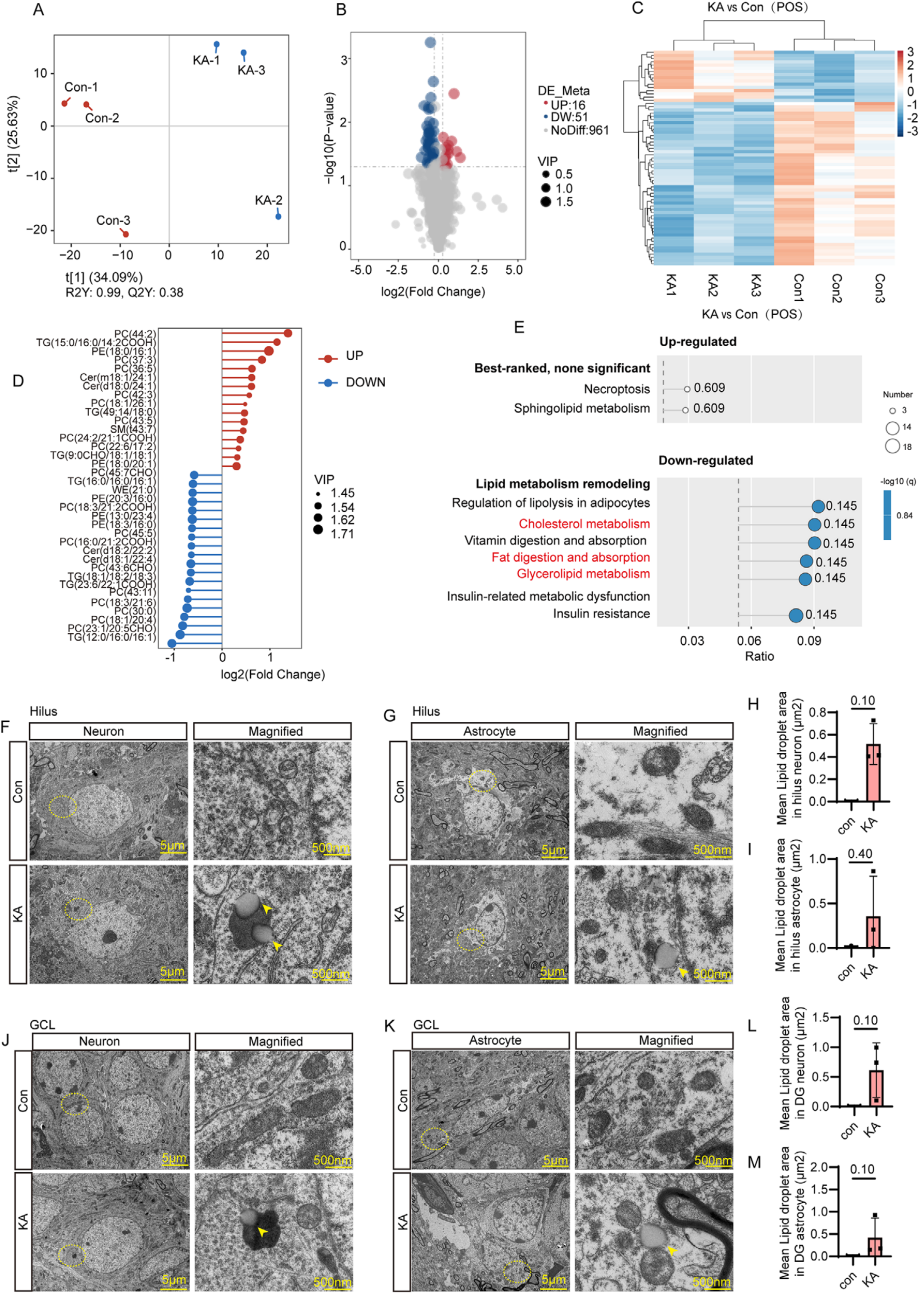
Exploratory hippocampal lipidomic differences and lipid droplet measurements 14 days after KA induction. (A) PLS‐DA score plot of hippocampal lipidomic profiles from KA‐treated and control mice in positive‐ion mode. Each point represents one biological sample (*n = 3* per group). R^2^Y = 0.99 and Q^2^Y = 0.38. (B) Volcano plot of differentially expressed lipids between Con and KA groups. Colors indicate upregulated (red) and downregulated (blue) lipid species, and point size reflects variable importance in projection (VIP) scores. (C) Heatmap of selected differentially expressed lipids in the positive ion mode (POS), showing distinct clustering patterns between Con and KA groups. (D) Top differentially altered lipid species, including phosphatidylcholine (PC), phosphatidylethanolamine (PE), triglycerides (TG), and ceramides (Cer), shown as log_2_ fold change with corresponding VIP scores (point size). Red and blue indicate increased and decreased abundance, respectively. Point size represents VIP scores. (E) KEGG pathway enrichment analysis of differential lipids. Upregulated and downregulated lipid sets were analyzed separately. The displayed upregulated pathways represent the highest‐ranked pathways, although none reached statistical significance after correction. Downregulated lipid‐associated pathways included cholesterol metabolism, vitamin digestion and absorption, fat digestion and absorption, glycerolipid metabolism, and insulin‐related pathways. Dot size indicates the number of mapped lipid species. Color encodes −log_10_(*q*), where *q* is the Benjamini–Hochberg‐adjusted *p* value; *q* values are labeled beside the points. (F,G) Representative transmission electron microscopy (TEM) images showing LD in neurons (F) and astrocytes (G) within the hippocampal hilus of Con and KA‐treated mice. Yellow arrows indicate LDs. Scale bars: 5 µm (main panels) and 500 nm (magnified insets). (H,I) Quantification of the mean total LD area per TEM image in neuronal (H) and astrocytic (I). (J,K) Representative TEM images showing LD (yellow dashed circles and arrows) in neurons (J) and astrocytes (K) within the hippocampal granule cell layer (GCL) of Con and KA‐treated mice. Scale bars: 5 µm (main panels) and 500 nm (magnified insets). (L,M) Quantification of mean total LD area per TEM image in GCL neurons (L) and astrocytes (M). Data in (H,I,L,M) are presented as mean ± SD, with individual dots representing individual biological animals. Statistical comparisons were performed using the two‐sided Mann–Whitney U test (n = *3* per group). Numbers above bars indicate exact non‐significant *P* values.

Given that triglycerides and other neutral lipids are major components of LDs [29], we next employed TEM to assess whether the observed lipid alterations were accompanied by ultrastructural changes in the hippocampus. LDs were detected across neuronal and astrocytic populations in KA‐treated mice, whereas control values were absent or minimal. In the hippocampal hilus, the mean LD area in neurons was 0.52 ± 0.19 µm^2^ in KA‐treated mice compared with 0 µm^2^ in controls (*n = 3* per group; Cohen's d = 3.88). In hilar astrocytes, the corresponding values were 0.36 ± 0.44 and 0.01 ± 0.02 µm^2^, respectively (Cohen's d = 1.14). Similarly, in the granule cell layer (GCL), the mean LD area was 0.61 ± 0.46 µm^2^ in neurons and 0.42 ± 0.44 µm^2^ in astrocytes from KA‐treated mice, whereas no LD area was detected in the corresponding control samples (Cohen's d = 1.84 and 1.44, respectively; *n = 3* per group). Group means and standard deviations calculated from mouse‐level TEM measurements are provided in Table S3. Together, these findings showed larger mean LD area measures in KA‐treated mice across the examined neuronal and astrocytic populations. Because these comparisons did not reach statistical significance and included only three mice per group, they should be interpreted as exploratory rather than evidence of increased LD accumulation in each cell population.

### 2.2 Reactive Astrocytes Exhibit Increased ABCA1 Expression During Epileptogenesis

To identify potential cellular mechanisms underlying seizure‐associated lipid remodeling, we next examined the expression of ABCA1, a key regulator of cellular cholesterol efflux and lipid transport [30]. Reanalysis of the astrocyte population in a publicly available human single‐nucleus RNA sequencing (snRNA‐seq) dataset revealed that ABCA1 expression was identified as a nominal candidate with higher expression in astrocytes from TLE patients compared with controls (Figure 2A), indicating a potential association with TLE‐related pathological processes. GO enrichment analysis (Figure 2B) showed that genes upregulated in TLE astrocytes were significantly associated with gliogenesis, glial cell differentiation, cholesterol biosynthesis, regulation of lipid metabolism, and regulation of neuronal differentiation and migration. In contrast, genes downregulated in TLE were enriched in processes related to synapse organization, regulation of synapse structure or activity. These results suggest that astrocytes undergo a transcriptional alteration associated with lipid metabolism and cellular remodeling in epilepsy‐associated hippocampal pathology. To further examine whether these observations were recapitulated in an experimental epilepsy model, we analyzed a mouse hippocampal scRNA‐seq dataset (GSE318570) derived from mouse hippocampi collected 4 days after KA intraperitoneal injection. Following clustering and annotation of major neural cell populations (Figure S1A,B), differential expression analysis identified *Abca1* as a nominal candidate gene with higher expression in astrocytes from KA‐treated mice compared with controls (Figure 2C). GO enrichment analysis revealed that genes upregulated in KA‐treated astrocytes were enriched in biological processes related to actin‐based movement and neuron projection regeneration, whereas downregulated genes were enriched in processes related to synapse organization and glutamatergic neuron differentiation (Figure 2D). Cell‐type expression profiles showed that *ABCA1/Abca1* was expressed in astrocytes in both the human and mouse datasets (Supplementary Figure S1C,D).

**FIGURE 2:**
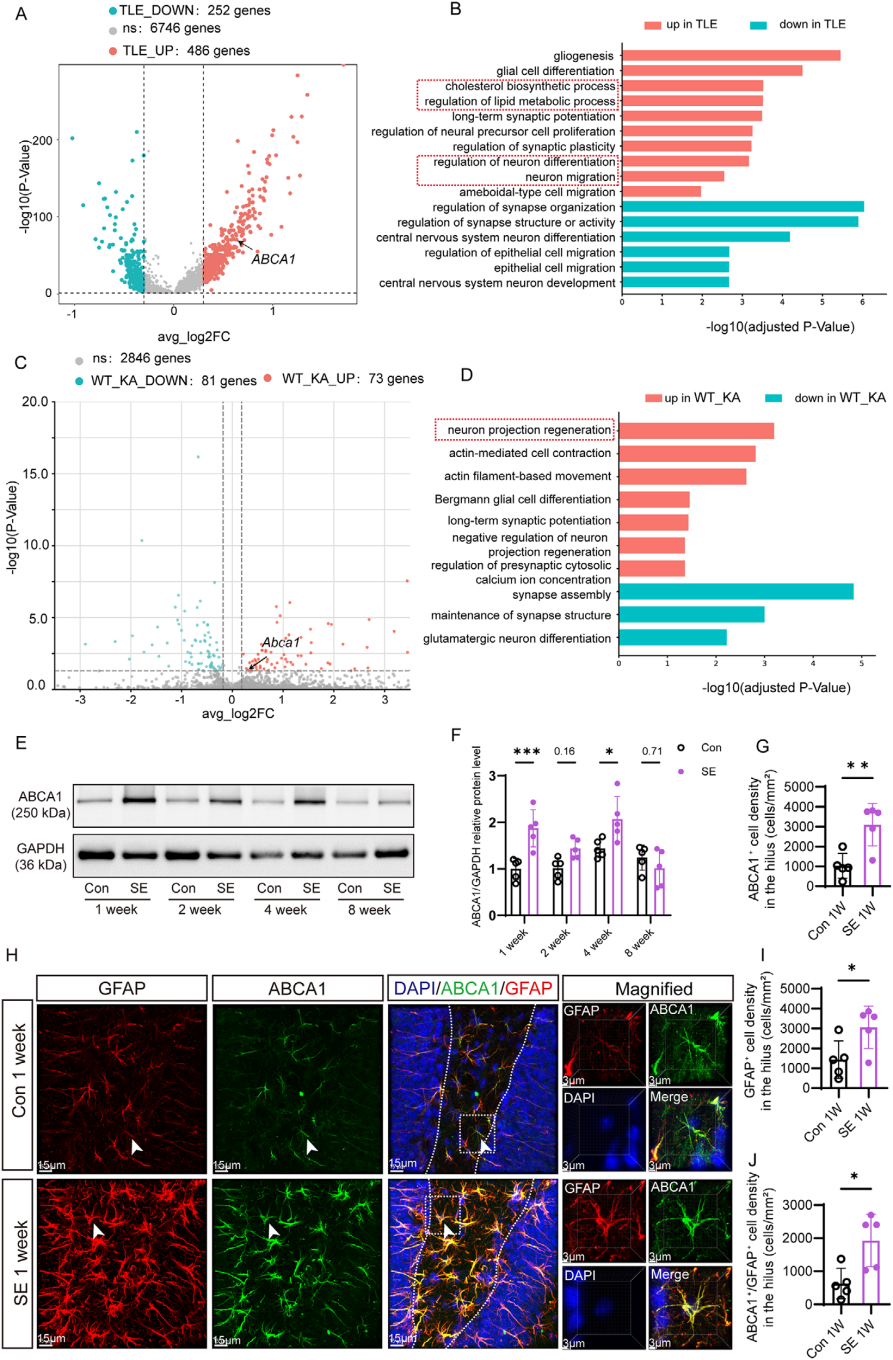
ABCA1 is dysregulated in astrocytes in temporal lobe epilepsy (TLE) and is associated with astrocytic responses after SE. (A) Volcano plot showing differentially expressed genes (DEGs) in astrocytes from TLE patients, indicating upregulated (TLE_UP, red) and downregulated (TLE_DOWN, cyan) genes compared with normal controls based on dataset GSE190453. *ABCA1* is highlighted as a nominal candidate gene with higher expression in TLE astrocytes. (ns, non‐significant). (B) Gene Ontology (GO) enrichment analysis of DEGs in astrocytes from TLE patients displaying top biological pathways associated with upregulated (up in TLE, red) and downregulated (down in TLE, cyan) genes. (C) Volcano plot showing DEGs in mouse astrocytes at 4 days after kainic acid (KA) intraperitoneal injection (dataset GSE318570), showing upregulated (WT_KA_UP, red) and downregulated (WT_KA_DOWN, cyan) genes compared with controls. *Abca1* is highlighted as a nominal candidate gene with higher expression in KA‐treated astrocytes. (D) GO enrichment analysis of upregulated (up in WT_KA, red) and downregulated (down in WT_KA, cyan) DEGs in mouse astrocytes from the KA model. (E) Western blot analysis of ABCA1 protein expression in the mouse hippocampus from control (Con) and status epilepticus (SE) groups at 1, 2, 4, and 8 weeks after SE. GAPDH served as a loading control. (F) Quantification of relative ABCA1 protein level normalized to GAPDH at 1, 2, 4, and 8 weeks after SE. (G) Quantification of ABCA1^+^ cell density in the hippocampal hilus at 1 week after SE. (H) Representative immunofluorescence images showing co‐staining of GFAP (astrocyte marker, red), ABCA1 (green), and DAPI (nuclei, blue) in the hippocampal hilus at 1week after SE. White arrowheads indicate representative GFAP+/ABCA1+ astrocytes. Scale bars: 15 µm (main panels) and 3 µm (magnified insets). (I,J) Quantification of GFAP^+^ cell density (I) and the density of ABCA1+/GFAP+ cells (J) in the hippocampal hilus at 1 week after SE. Data are presented as mean ± SD. Western blot data were analyzed using two‐way ANOVA followed by Sidak's multiple‐comparisons test. Immunofluorescence data were analyzed using two‐sided unpaired Welch's t‐tests. *n = 5* per group. ^*^ *p* < 0.05, ^**^ *p* < 0.01, ^***^ *p* < 0.001. Numbers above bars indicate exact non‐significant *P* values.

Protein‐level validation using our KA‐induced SE mouse model demonstrated that hippocampal ABCA1 protein levels were significantly elevated at 1 and 4 weeks post‐SE compared with the corresponding controls, whereas the differences at 2 and 8 weeks did not reach statistical significance (Cohen's d = 1.98 and 0.67, respectively; Figure 2E,F). Immunofluorescence analysis revealed a significantly increased density of ABCA1^+^ cells in the hippocampal hilus at 1 week post‐SE compared with controls (Figure 2G,H). This increase was accompanied by a higher density of GFAP^+^ astrocytes (Figure 2H,I). Notably, ABCA1 immunoreactivity was observed in GFAP^+^ astrocytes, and the density of ABCA1^+^/GFAP^+^ double‐positive cells was also significantly increased at 1 week post‐SE (Figure 2H,J). Together, these findings demonstrate that ABCA1/Abca1 was identified as a nominal astrocyte‐associated candidate in both human TLE and KA‐treated mouse datasets. This transcriptomic pattern, together with the increased density of ABCA1^+^ /GFAP^+^ astrocytic profiles in the hilus after SE, is consistent with a potential involvement of ABCA1 in epilepsy‐associated astrocytic responses.

### 2.3 Astrocytic APOE and ABCA1 are Increased in Distinct Experimental Epilepsy Models

Building upon the observation of hippocampal lipid alterations (Figure 1) and astrocytic ABCA1 upregulation (Figure 2), we next examined whether APOE‐associated lipid transport pathways were also altered during epileptogenesis. APOE is the primary carrier involved in lipid transport and has been proven to mediate the transfer of lipids from neurons to astrocytes [13]. Western blot analysis in the KA‐induced model (Figure 3A,B) showed that hippocampal APOE protein levels were significantly elevated at 2 weeks post‐SE compared with controls, while differences at the other examined time points were not statistically significant. Furthermore, immunofluorescence staining at 1 week post‐SE revealed a marked increase in the percentage of APOE^+^/GFAP^+^ cellular profiles among GFAP^+^ cells within the hippocampal hilus (Figure 3C,E), despite the absence of a significant increase in bulk hippocampal APOE protein at this time point. High‐resolution imaging further showed APOE and ABCA1 immunoreactivity spatially associated with GFAP‐positive astrocytic profiles (Figure 3E). Quantitative analysis confirmed a significant increase in the percentage of ABCA1^+^/APOE^+^ astrocytes among the total hilus GFAP^+^ population in the SE group (Figure 3D), supporting an astrocyte‐associated APOE/ABCA1 response during epileptogenesis.

**FIGURE 3:**
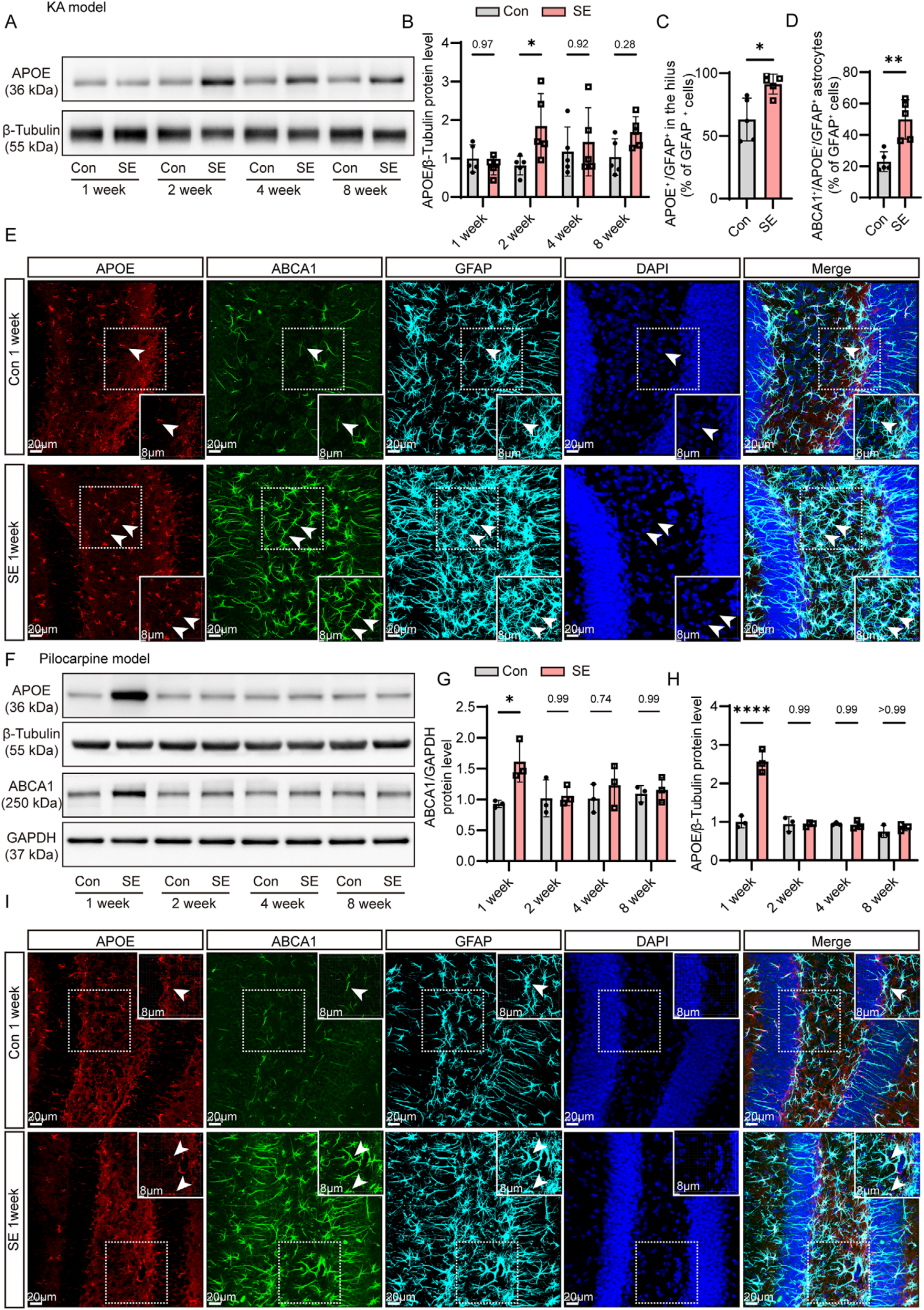
ABCA1 and APOE expression is altered in hippocampal astrocytes in KA‐ and pilocarpine‐induced epilepsy models. (A,B) Western blot analysis (A) and quantification of APOE/β‐Tubulin (B) in the mouse hippocampus at 1, 2, 4, and 8 weeks after KA‐induced status epilepticus (SE) (*n* = 5 per group). (C,D) Quantification of the percentage of APOE‐expressing astrocytes (APOE^+^/GFAP^+^ double‐positive, C) and ABCA1/APOE‐co‐expressing astrocytes (ABCA1^+^/APOE^+^/GFAP^+^ triple‐positive, D) relative to total GFAP^+^ cells in the hippocampal hilus at 1 week after KA‐induced SE (*n* = 5 per group). (E) Representative immunofluorescence images showing the expression of APOE (red), ABCA1 (green), and GFAP (astrocyte marker, cyan) in the hippocampus at 1 week after KA‐induced SE. Nuclei were counterstained with DAPI (blue). White dashed boxes indicate the regions shown in the magnified insets, and white arrowheads indicate APOE and ABCA1 immunoreactivity associated with GFAP^+^ astrocytic profiles. Scale bars: 20 µm (main panels) and 8 µm (magnified insets). (F–H) Western blot analysis of ABCA1 and APOE expression in the pilocarpine‐induced epilepsy model (F), with quantification of relative ABCA1/GAPDH (G) and APOE/β‐Tubulin (H) protein levels at different time points (*n* = 3 per group). (I) Representative multiplex immunofluorescence images showing expression of APOE (red), ABCA1 (green), GFAP (cyan) and DAPI (blue) at 1 week after pilocarpine‐induced SE. White dashed boxes indicate the regions shown in the magnified insets, and white arrowheads indicate APOE and ABCA1 immunoreactivity associated with GFAP^+^ astrocytic profiles. Scale bars: 20 µm (main panels) and 8 µm (magnified insets). Data are presented as mean ± SD. Western blot data were analyzed using two‐way ANOVA followed by Sidak's multiple‐comparisons test. Immunofluorescence data were analyzed using two‐sided unpaired Welch's t‐tests. ^*^ *p* < 0.05, ^**^ *p* < 0.01, ^****^ *p* < 0.0001. Numbers above bars indicate exact non‐significant *P* values.

Because ABCA1 regulates APOE lipidation and secretion [31], their coordinated upregulation in astrocytes suggests an enhanced astrocyte‐associated lipid transport capacity following seizure induction. To determine whether this response represents a common feature of epilepsy rather than a KA model‐specific effect, we next examined a pilocarpine‐induced epilepsy model. Consistent with observations from the KA model, Western blot analysis revealed significant increases in both ABCA1 and APOE protein levels in the hippocampus at 1 week post‐pilocarpine SE (Figure 3F–H). Multiplex immunofluorescence further showed APOE and ABCA1 immunoreactivity associated with GFAP^+^ astrocytes (Figure 3I), supporting an astrocytic contribution to this response.

The two models differed in their recorded behavioral seizure burden, induction efficacy, and mortality profiles (Figure S1E–G and Table S2). Among successfully induced mice, the KA cohort showed a significantly higher mean frequency of Racine stage IV or higher seizures than the pilocarpine cohort (4.41 ± 1.97 vs. 2.54 ± 0.66 episodes per mouse, p < 0.01), alongside a greater cumulative behavioral burden (21.66 ± 9.79 vs. 12.38 ± 2.82, p < 0.01). Furthermore, the KA model exhibited a numerically higher induction success rate (87.88% vs. 54.17%) and a numerically lower mortality rate during induction (6.06% vs. 20.83%) compared to the pilocarpine model. Because continuous EEG was not recorded during the acute induction period, the duration of electrographic SE could not be determined or directly compared between models. Therefore, the molecular similarities observed between models should be interpreted as findings observed across two distinct chemoconvulsant paradigms, rather than as evidence that the two models produced identical status epilepticus severity or duration.

To further assess the cellular distribution of APOE transcripts, we analyzed snRNA‐seq datasets, which confirmed that APOE/Apoe transcripts are predominantly enriched in astrocytic populations in both human and mouse brains (Figure S2A,B). To further characterize the temporal dynamics and cellular distribution of APOE expression, we examined the hippocampal hilus at 2 and 8 weeks after KA‐induced SE (Figure S2C–E). At 2 weeks post‐SE, GFAP^+^ astrocyte density and the percentage of APOE^+^ astrocytes among GFAP^+^ cells were significantly increased compared with controls (Figure S2D,E), and APOE immunoreactivity was observed in association with GFAP‐labelled astrocytes (Figure S2C). At 8 weeks post‐SE, neither measure differed significantly between the SE and control groups (Figure S2D,E). Additionally, at 1 week after SE, hilar NeuN^+^ neuronal density was significantly reduced, whereas S100β^+^ astrocyte density was significantly increased (Figure S2F–H), further supporting SE‐associated cellular remodeling in the hippocampal hilus. Taken together, these findings demonstrate that epilepsy is associated with an astrocyte‐associated ABCA1/APOE response across two in vivo models. Although differences in seizure burden and mortality between models may contribute to the magnitude of these changes, the consistent astrocytic localization supports the involvement of astrocyte‐associated lipid handling during epileptogenesis.

### 2.4 Ectopically Located Neurons were Spatially Associated with ABCA1‐Positive Cells and ABCA1/GFAP‐Associated Astrocytic Profiles in the Hippocampal Hilus

Because increased ABCA1‐associated astrocytic labelling was observed in the hippocampal hilus (Figure 2H), and ectopic migration of dentate granule cells into this region represents a well‐recognized pathological feature associated with epileptogenesis and SRS [20], we next investigated whether ectopically located neuronal populations were associated with nearby PLIN2‐positive puncta. Immunofluorescence analysis showed that following SE, the densities of Prox1^+^ granule cells and DCX^+^ immature neurons were significantly increased within the hippocampal hilus at 1 week (Figure 4A–D). Time‐course analyses further showed increased hilar Prox1^+^ cell density at 1 and 2 weeks, whereas the difference at 4 weeks did not reach statistical significance (Cohen's d = 2.39) (Figure S3D). Co‐staining with the lipid droplet‐associated protein PLIN2 showed PLIN2^+^ puncta in close spatial association with Prox1^+^ and DCX^+^ profiles in the hilus at 1 week post‐SE (Figure S3A,B), suggesting a spatial association between these ectopic neuronal populations and nearby PLIN2‐positive puncta.

**FIGURE 4:**
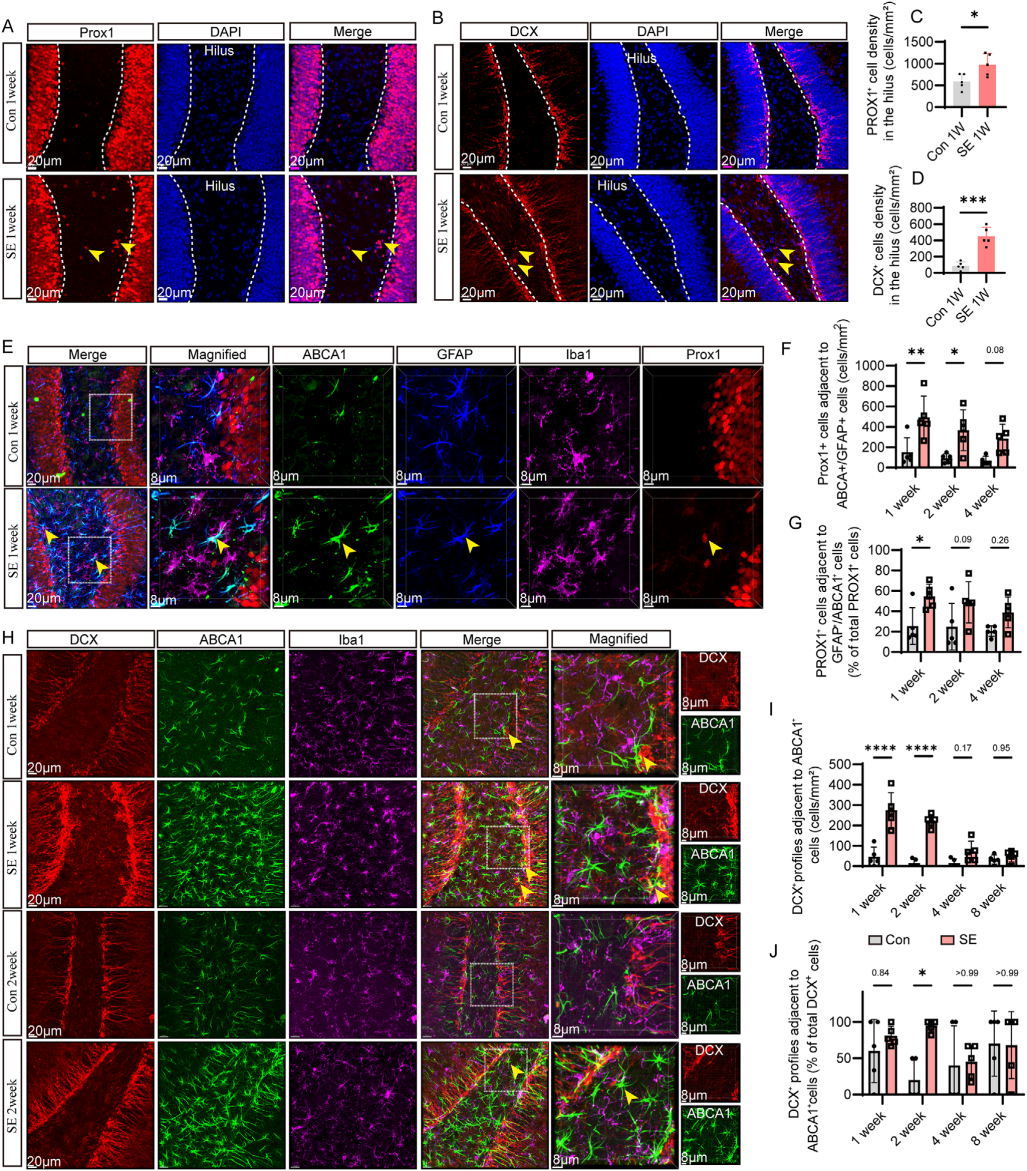
Status epilepticus induces ectopic neuronal accumulation in the hippocampal hilus and alters the spatial association of ectopic neurons with ABCA1^+^ cells, including ABCA1/GFAP‐associated astrocytic profiles. (A,B) Representative immunofluorescence images of Prox1 (A, red) or DCX (B, red) and DAPI (blue) in the hippocampal hilus at 1 week after SE. White dashed lines delineate the hilar boundary. Yellow arrowheads indicate ectopically located Prox1^+^ granule cells or DCX^+^ immature neurons within the hilus. Scale bars: 20 µm (main panels), 8 µm (magnified insets). (C,D) Quantification of the density of Prox1^+^ cells (C) and DCX^+^ cells (D) in the hippocampal hilus at 1 week after SE (n = 5 per group). (E) Representative immunofluorescence images of ABCA1 (green), GFAP (blue), Iba1 (magenta), and Prox1 (red) in the hippocampal hilus of control mice and mice at 1 week after SE. Yellow arrowheads indicate representative ectopic Prox1^+^ cells spatially adjacent to ABCA1^+^/GFAP^+^ astrocytes. Scale bars: 20 µm (main panels), 8 µm (magnified insets). (F, G) Density of Prox1^+^ cells adjacent to ABCA1^+^/GFAP^+^ cells (F) and the proportion of total Prox1^+^ cells adjacent to ABCA1^+^/GFAP^+^ cells (G) in the hilus at 1, 2, and 4 weeks after SE. (*n = 5* per group). (H) Representative immunofluorescence images of DCX (red), ABCA1 (green), and Iba1 (magenta) in the hippocampal hilus at 1 and 2 weeks after SE. White dashed boxes indicate the regions shown in the magnified panels, and yellow arrowheads indicate representative DCX^+^ profiles adjacent to ABCA1^+^ cells. Scale bars: 20 µm (main panels), 8 µm (magnified insets). (I,J) Density of DCX^+^ profiles adjacent to ABCA1^+^ cells (I) and the proportion of DCX^+^ profiles adjacent to ABCA1^+^ cells among total DCX^+^ profiles (J) at 1, 2, 4, and 8 weeks after SE. (*n = 5* per group). Data are presented as mean ± SD. Panels C and D were analyzed using two‐sided unpaired Welch's t‐tests. Panels F, G, I, and J were analyzed by ordinary two‐way ANOVA with Sidak's multiple‐comparisons test. ^*^ *p* < 0.05, ^**^ *p* < 0.01, ^***^ *p* < 0.001, ^****^ *p* < 0.0001. Numbers above bars indicate exact non‐significant *P* values.

Given the concurrent increase in ABCA1/GFAP‐associated astrocytic labelling observed in the hilus (Figure 2H), we next examined whether ABCA1‐positive cells, including GFAP‐positive astrocytic profiles, were spatially associated with these ectopic neuronal populations. Immunofluorescence staining revealed that Prox1‐labelled ectopic granule cells were adjacent to ABCA1^+^/GFAP^+^ astrocytic profiles within the hilus (Figure 4E and Figure S3C, yellow arrowheads). Quantitative analysis further demonstrated an increased density of Prox1^+^ cells adjacent to ABCA1^+^/GFAP^+^ astrocytes at 1 and 2 weeks post‐SE. At 4 weeks, the difference did not reach statistical significance (Cohen's d = 2.06) (Figure 4F). The percentage of Prox1^+^ cells adjacent to these astrocytes among total hilar Prox1^+^ cells was significantly increased at 1 week; the differences at 2 and 4 weeks did not reach statistical significance (Cohen's d = 1.11 and 1.62, respectively) (Figure 4G). Similarly, DCX^+^ profiles were observed adjacent to ABCA1^+^ cells (Figure 4H and Figure S3I). The density of DCX^+^ profiles adjacent to ABCA1^+^ cells was significantly increased at 1 and 2 weeks post‐SE (Figure 4I). After normalization to the total DCX^+^ population, the percentage of DCX^+^ profiles adjacent to ABCA1^+^ cells was significantly increased only at 2 weeks, but not at 1, 4, or 8 weeks post‐SE (Figure 4J). Because GFAP was not included in the DCX/ABCA1/Iba1 staining panel, these profiles are reported as ABCA1^+^ cells rather than ABCA1^+^ astrocytes. The prominent ABCA1‐positive, DCX‐negative, and Iba1‐negative process‐bearing profiles visible in this panel were not assigned to a neuronal cell population. Their morphology, together with the ABCA1/GFAP staining shown in Figure 4E, is consistent with an astrocytic contribution to the ABCA1 signal; however, this interpretation was not used to classify individual cells or for quantitative analysis.

Because microglial activation represents another prominent feature of the epileptic hippocampal microenvironment, we next examined microglial density and the spatial relationship between microglia and ectopic Prox1^+^ cells in this region [32]. Following SE, Iba1^+^ microglial density was significantly increased at 1 and 2 weeks, but not at 4 weeks (Figure S3F). GFAP^+^ astrocyte density was significantly increased at 1 week, whereas the differences at 2 and 4 weeks were not statistically significant (Cohen's d = 1.48 and 0.08, respectively) (Figure S3E). The Cohen's d values reported for nonsignificant comparisons are descriptive and do not establish group differences at those time points. Despite the increase in microglial density, the percentage of Prox1^+^ cells adjacent to Iba1^+^ cells did not differ significantly between SE and control groups at any examined time point (Figure S3G). In contrast, the percentage of DCX^+^ profiles adjacent to Iba1^+^ cells was increased at 1 week after SE but did not differ significantly at later time points (Figure S3H). Thus, the early increase in Iba1+ microglial density was not accompanied by a sustained increase in the proportion of ectopic neuronal profiles adjacent to microglia. Together, these findings demonstrate that ectopically located granule cells are increased in density in the hilus after SE and exhibit temporally dynamic spatial associations with ABCA1^+^ cells, including ABCA1^+^/GFAP^+^ astrocytic profiles. Together with the qualitative observation of PLIN2‐positive puncta near ectopic neuronal profiles, these observations motivate further investigation of a possible relationship between ectopic neuronal populations and the local astrocyte‐associated lipid metabolic environment during epileptogenesis.

### 2.5 Bexarotene Pretreatment Prolonged KA‑Induced Seizure Latency and Reduced Ectopic Neuronal Accumulation in the Hippocampal Hilus

Although hippocampal ABCA1 protein levels were increased at selected time points after SE, these changes occurred alongside the lipid alterations and pathological remodeling described above. Therefore, pharmacological enhancement of this pathway was pursued using the RXR agonist bexarotene [33]. Previous studies in Alzheimer's disease models have reported that bexarotene increases ABCA1/APOE expression and lipidation, facilitates cholesterol transport, and improves cognitive deficits [34]. To investigate whether pharmacological activation of the RXR signaling affects epilepsy‐related phenotypes, we employed a bexarotene pretreatment strategy prior to KA‐SE. The experimental timelines for both in vivo and in vitro studies are shown in Figure 5A,B. Western blot analysis confirmed increased hippocampal ABCA1 protein expression following bexarotene pretreatment compared with vehicle controls (Figure 5F,G; p < 0.05). No significant differences in body weight were observed between groups (Figure 5H). All mice initially received KA at 25 mg/kg. One Bex‐pretreated mouse received a prespecified supplemental dose of 5 mg/kg after failing to develop a Racine stage IV or higher seizure during the initial 60‐min observation period; this mouse subsequently remained unsuccessful and was excluded from chronic EEG analysis (Figure 5I and Figure S5A). One of the six Bex‐pretreated mice did not exhibit Racine stage IV or higher seizures, whereas all six vehicle‐pretreated mice exhibited such seizures (Figure 5J). Among mice exhibiting qualifying seizures, behavioral assessment revealed that bexarotene pretreatment significantly prolonged the latency to the first KA‐induced seizure (Figure 5K; p < 0.05). After the first qualifying seizure, animals were monitored for an additional 60 min according to the predefined protocol, after which seizure activity was terminated with diazepam. Six mice were initially included in each group. Two vehicle‐pretreated mice and one Bex‐pretreated mouse died, leaving four and five survivors, respectively. The Bex‐pretreated mouse without qualifying seizures survived but was excluded from chronic EEG monitoring. Consequently, chronic EEG recordings were obtained from four mice per group (Figure S5A). Within the analyzed cohort, neither the number of Racine stage≥IV seizures nor the cumulative behavioral seizure burden differed significantly between groups during the standardized observation window (Figure S5B,C). Chronic EEG recordings showed that bexarotene pretreatment was associated with a significantly lower frequency of spontaneous seizures during the chronic monitoring period (Figure 5L,M), whereas seizure duration and average spectral power were numerically lower but did not differ significantly (Figure 5N,O). Because continuous EEG recordings were not obtained throughout the acute induction period, these data do not establish an effect of bexarotene on the duration of electrographic SE. Accordingly, the lower frequency of chronic spontaneous seizures should be interpreted as an association observed among surviving mice with qualifying behavioral seizures, rather than as evidence that bexarotene shortened SE or definitively prevented epileptogenesis. Together, these findings show that bexarotene pretreatment delayed the onset of severe behavioral seizures and was associated with a lower frequency of chronic spontaneous seizures among surviving, successfully induced mice.

**FIGURE 5:**
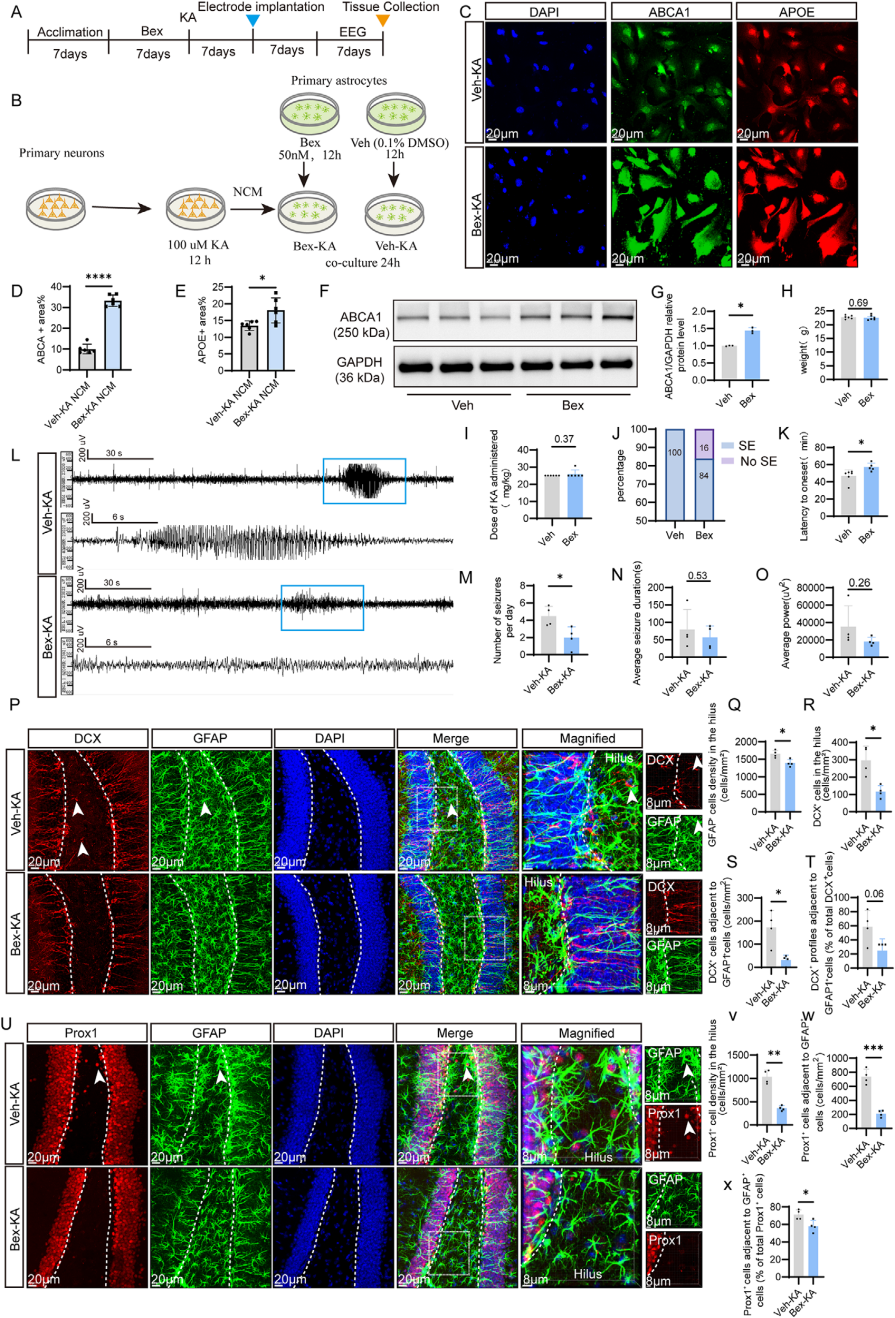
Bexarotene pretreatment increases ABCA1 expression and is associated with delayed acute seizure onset, lower chronic seizure frequency, and reduced hilar ectopic neuronal accumulation after KA administration. (A) Timeline of the in vivo 7‐day bexarotene‐pretreatment experiment. Mice received Bex or vehicle for 7 days before KA administration. Electrodes were implanted 7 days after KA administration, followed by a 7‐day recovery period and 7 days of EEG monitoring before tissue collection. (B) Schematic of the in vitro neuronal conditioned‐medium (NCM) paradigm. Primary neurons were exposed to KA (100 µM, 12 h), after which NCM was collected. Primary astrocytes were pretreated with Bex (50 nM) or vehicle for 12 h and subsequently exposed to NCM for 24 h. (C) Representative immunofluorescence images of primary astrocytes exposed to Veh‐KA NCM or Bex‐KA NCM, showing DAPI (nuclei, blue), ABCA1 (green), and APOE (red). Scale bars: 20 µm. (D,E) Quantification of ABCA1^+^ area fraction (D) and APOE^+^ area fraction (E) in cultured astrocytes exposed to Veh‐KA NCM or Bex‐KA NCM (*n = 5* per group). (F,G) Representative Western blots (F) and quantification of hippocampal ABCA1 protein levels normalized to GAPDH (G) in Veh‐ and Bex‐pretreated mice (*n = 3* per group). (H,I) Body weight at the end of pretreatment (H), KA dose administered (I) in Veh‐ and Bex‐pretreated mice (*n = 6* per group). (J) Proportions of mice that developed qualifying behavioral SE or did not develop qualifying Racine stage IV or higher seizures after KA administration (*n = 6* per group). (K) Latency to the first KA‐induced Racine stage IV or higher seizures in Veh‐pretreated (*n = 6*) and Bex‐pretreated mice (*n = 5*). (L–O) Representative chronic EEG traces (L) and quantification of number of seizures per day (M), average seizure duration (N), and average seizure power (O) in the Veh‐KA and Bex‐KA groups. (*n = 4*). (P–T) Representative immunofluorescence images (P) and quantification of GFAP^+^ cell density (Q), DCX^+^ cell density (R), DCX^+^ cells adjacent to GFAP^+^ astrocytes (S), and the proportion of DCX^+^ cells adjacent to GFAP^+^ astrocytes (T) within the hippocampal hilus. White arrowheads indicate ectopically located DCX^+^ cells, and dashed lines indicate the hilar boundary. Scale bars: 20 µm (main panels) and 8 µm (magnified images). (U–X) Representative immunofluorescence images (U) and quantification of Prox1^+^ cell density (V), Prox1^+^ cells adjacent to GFAP^+^ astrocytes (W), and the proportion of Prox1^+^ cells adjacent to GFAP^+^ astrocytes (X) within the hippocampal hilus. White arrowheads indicate ectopically located Prox1^+^ cells. Scale bars: 20 µm (main panels) and 8 µm (magnified images). Data are presented as mean ± SD. Panels D, E, G–I, K, M–O, and Q–X were analyzed using two‐sided unpaired Welch's t‐tests. Panel J was analyzed using Fisher's exact test. For the hilar immunofluorescence analyses, *n = 4* mice per group. ^*^ *p* < 0.05, ^**^ *p* < 0.01, ^***^ *p* < 0.001, ^****^ *p* < 0.0001. Numbers above bars indicate exact non‐significant *P* values.

Because ectopic migration of dentate granule cells into the hippocampal hilus represents a hallmark structural abnormality associated with epileptogenesis [18], we next examined whether bexarotene pretreatment is associated with changes in this pathological feature. Compared with Veh‐treated epileptic mice, Bex‐treated mice exhibited reduced densities of hilar DCX^+^ immature neurons and ectopically located Prox1+ granule cells (Figure 5P,R,U,V). Quantitative analysis further demonstrated that pretreatment with bexarotene significantly decreased the GFAP^+^ astrocyte density (Figure 5Q). The density of DCX^+^ cells adjacent to GFAP^+^ cells was also reduced (Figure 5S), whereas the percentage of DCX^+^ profiles adjacent to GFAP^+^ cells did not reach statistical significance (Cohen's d = 1.68, p = 0.06) (Figure 5T). The observed effect size for this percentage comparison is descriptive and does not establish a treatment effect. Both the density and percentage of Prox1^+^ cells adjacent to GFAP^+^ cells were significantly decreased (Figure 5W,X). These findings suggest that bexarotene pretreatment was associated with lower hilar densities of ectopically located neuronal populations and GFAP^+^ astrocytes.

To further examine cell‐type‐specific lipid transport responses following bexarotene treatment, we next evaluated APOE expression in primary neuronal and astrocyte cultures. Immunofluorescence and mRNA analyses revealed that KA exposure significantly increased APOE‐positive area and Apoe mRNA levels in neuronal cultures (Figure S4A–C). In astrocytes, KA exposure did not significantly alter total APOE‐positive area but significantly increased the ABCA1/APOE overlapping area (Figure S4D–F). Because astrocytes respond dynamically to neuron‐derived lipid signals following hyperexcitability, we further examined astrocytic responses using NCM derived from KA‐treated neurons (Figure 5B). Primary astrocytes pretreated with bexarotene and subsequently exposed to KA‐NCM showed significantly increased ABCA1‐positive and APOE‐positive immunoreactive areas compared with vehicle‐pretreated astrocytes exposed to the same conditioned medium (Figure 5C–E). GFAP‐positive area did not differ significantly between groups (Figure S4G). In addition, bexarotene pretreatment significantly increased the areas of overlapping ABCA1/APOE/GFAP and PLIN2/APOE/GFAP signals in astrocytes exposed to KA‐NCM (Figure S4H,J). These area‐based measurements describe altered immunoreactivity and do not establish lipid‐droplet abundance, definitive cellular co‐localization, or lipid transport capacity.

To further assess neuronal and glial changes associated with bexarotene pretreatment in vivo, we examined NeuN‐, S100β‐, and Iba1‐labelled cells in the hippocampal hilus (Figure S5D–L). Immunofluorescence analysis revealed that bexarotene treatment significantly increased NeuN+ neuronal density and decreased S100β+ cell density (Figure S5D–F), whereas Iba1+ microglial density was not significantly altered (Figure S5G). Morphological analysis further showed that bexarotene pretreatment significantly reduced microglial soma size (Figure S5H,J), while branch count, mean branch length, and maximum branch length did not differ significantly between groups (Cohen's d = 1.59 and 1.77 for mean and maximum length, respectively) (Figure S5I,K,L). Thus, a difference was detected in soma size, but the other measured microglial endpoints did not reach statistical significance. The reduction in S100β^+^ cell density was also consistent with the lower GFAP^+^ astrocyte density observed in Figure 5Q. Together, these findings demonstrate that pharmacological activation of RXR signaling enhances ABCA1 expression, delays seizure onset, and was associated with lower chronic spontaneous seizure frequency and altered neuronal and glial measures among surviving mice with qualifying behavioral seizures. These findings support further investigation of RXR‐ and astrocyte‐associated lipid transport pathways in epilepsy‐associated hippocampal remodeling.

### 2.6 Post‐SE Bexarotene Administration was Associated with Reduced Seizure Frequency and Lower Densities of Ectopically Located Neuronal Populations in the Hippocampal Hilus

To evaluate whether pharmacological activation of RXR signaling was associated with changes in chronic epilepsy‐related phenotypes, bexarotene was administered during the recovery period after KA‑induced SE. The experimental timeline is shown in Figure 6A. No between‐group difference in body weight was detected at KA administration, immediately before bexarotene treatment, or at the end of treatment (Figure 6B,C). In this cohort (n = 4 mice per group), EEG recording showed that bexarotene‐treated mice had a significantly lower frequency of spontaneous seizures during the chronic phase than vehicle‐treated mice (Figure 6D,E; P <0.01), whereas seizure duration and average spectral power did not differ significantly between groups (Figure 6F,G). These findings indicate that post‐SE administration of bexarotene was associated with a lower spontaneous seizure frequency during the monitored period.

**FIGURE 6:**
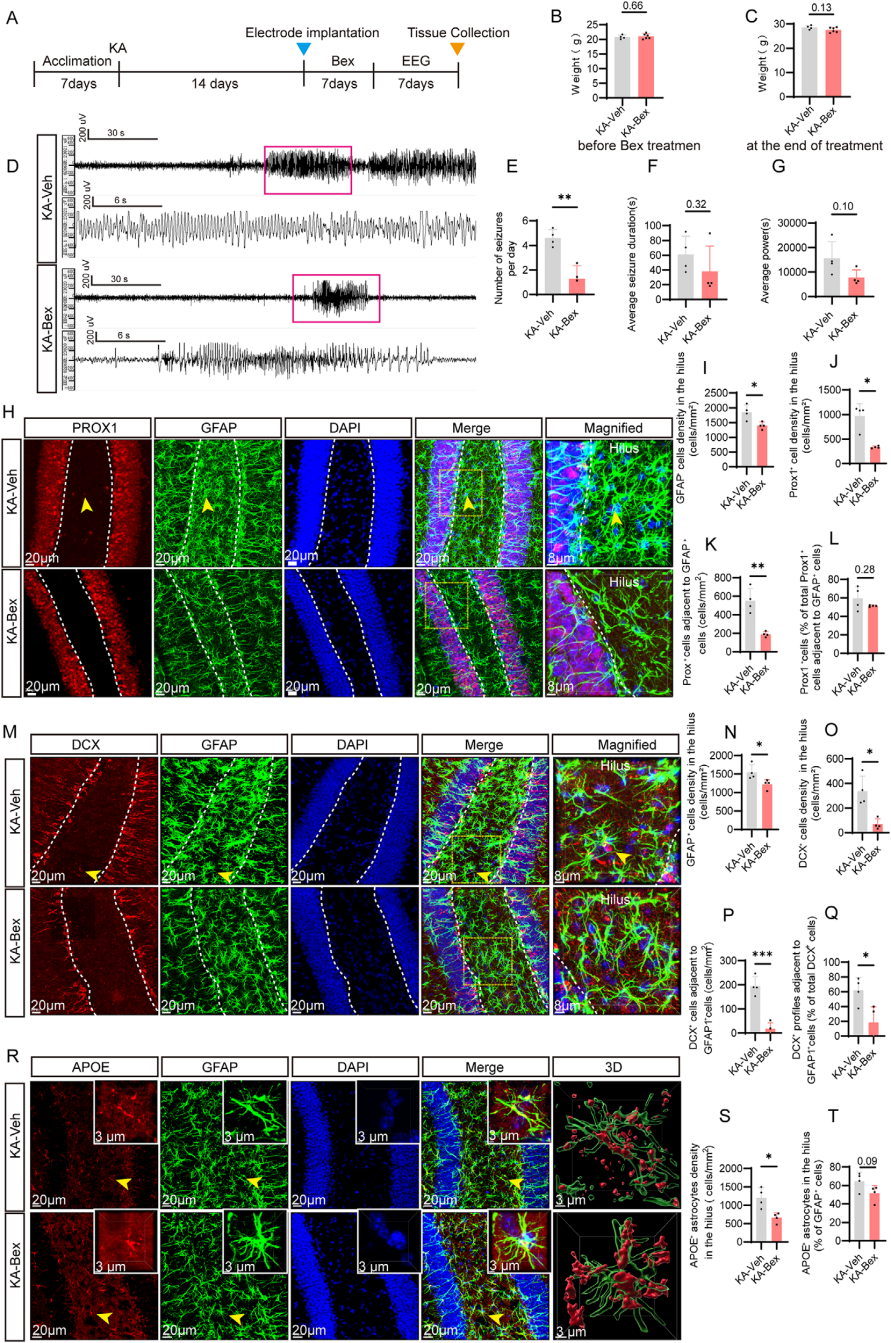
Post‐SE bexarotene treatment is associated with lower chronic seizure frequency and reduced densities of ectopic neurons and APOE‐positive astrocytic profiles in the hippocampal hilus after KA‐induced SE. (A) Schematic timeline of the experiment procedure, including acclimation, KA administration, electrode implantation, Bex treatment, EEG recording, and tissue collection. (B,C) Body weight of mice before Bex administration (B) and after 1 week of vehicle (Veh) or Bex treatment (C). (KA‐Veh, *n = 4*; KA‐Bex, *n = 6*). (D–G) Representative EEG traces (D) and quantification of number of seizures per day (E), average seizure duration (F), and average power (G) in KA‐Veh and KA‐Bex groups. (*n* = 4 per group). Boxed regions in the upper traces are shown at higher temporal resolution below. (H–L) Representative immunofluorescence images of Prox1 (red), GFAP (green), and DAPI (blue) in the dentate gyrus at 4 weeks after KA administration (H), with quantification of hilar GFAP^+^ astrocyte density (I), Prox1^+^ cell density (J), Prox1^+^ cells adjacent to GFAP^+^ astrocytes (K), and their proportion among total hilar Prox1^+^ cells (L). Yellow arrowheads indicate ectopically located Prox1^+^ cells. Scale bars: 20 µm (main panels) and 8 µm (magnified images) (*n* = 4 per group). (M–Q) Representative immunofluorescence images of DCX (red), GFAP (green), and DAPI (blue) in the dentate gyrus at 4 weeks after KA administration (M), with quantification of hilar GFAP^+^ astrocyte density (N), DCX^+^ cell density (O), DCX^+^ cells adjacent to GFAP^+^ astrocytes (P), and their proportion among total hilar DCX^+^ cells (Q). Yellow arrowheads indicate ectopically located DCX^+^ cells. Scale bars: 20 µm (main panels) and 8 µm (magnified images) (*n* = 4 per group). (R–T) Representative images and three‐dimensional reconstructions of APOE (red), GFAP (green), and DAPI (blue) in the hippocampal hilus at 4 weeks after KA administration (R), with quantification of APOE^+^ astrocytic‐profile density (S) and the proportion of APOE^+^ astrocytic profiles among total GFAP^+^ astrocytic profiles (T). Yellow arrowheads indicate APOE immunoreactivity associated with GFAP^+^ astrocytic profiles. Scale bars: 20 µm (main panels) and 3 µm (magnified images) (*n* = 4 per group). Data are presented as mean ± SD. Quantitative panels B, C, and E–G, I–L, N–Q, and S–T were analyzed using two‐sided unpaired Welch's t‐tests. ^*^ *p* < 0.05, ^**^ *p* <0.01, ^***^ *p* <0.001. Numbers above bars indicate exact non‐significant *P* values.

Because ectopic migration of dentate granule neurons into the hippocampal hilus represents a persistent structural abnormality associated with chronic epileptogenesis, we next examined whether therapeutic bexarotene administration modulates this pathology. Immunofluorescence analysis demonstrated that bexarotene treatment significantly reduced the densities of ectopically located Prox1^+^ granule cells and DCX^+^ immature neurons in the hippocampal hilus (Figure 6H,J,M,O). In parallel, bexarotene treatment was associated with a lower GFAP^+^ astrocyte density in the same region (Figure 6I,N). Furthermore, both the density of DCX^+^ cells adjacent to GFAP^+^ cells and the percentage of adjacent DCX^+^ cells among total hilar DCX^+^ cells were significantly decreased (Figure 6P,Q). The density of Prox1^+^ cells adjacent to GFAP^+^ cells was also significantly reduced, whereas the corresponding percentage among total hilar Prox1^+^ cells did not differ significantly between groups (Figure 6K,L). These findings indicate reduced ectopic neuronal accumulation and changes in neuronal–astrocytic spatial associations following treatment. Given the previously observed association between astrocytic lipid‐related signals and ectopic neuronal populations, we further assessed APOE‐associated changes in the hilus. Immunofluorescence analysis and three‐dimensional reconstructions illustrated the spatial association of APOE signals with GFAP+ astrocytes (Figure 6R). Quantification showed a significant reduction in APOE^+^ astrocyte density (Figure 6S), whereas the percentage of APOE^+^ astrocytes among GFAP^+^ cells did not differ significantly between groups (Cohen's d = 1.43, p = 0.09) (Figure 6T). The effect‐size estimate for this percentage comparison is descriptive and does not establish a treatment effect. These findings suggest that post‐SE bexarotene treatment was associated with a lower density of APOE^+^ astrocytes in the epileptic hippocampus.

To further evaluate glial and neuronal responses to therapeutic bexarotene administration, we examined additional cellular markers in the hippocampal hilus (Figure S6). Immunofluorescence staining demonstrated that bexarotene treatment significantly reduced S100β^+^ cell density (Figure S6A,B; p < 0.05), whereas NeuN^+^ neuronal and Iba1^+^ microglial densities were not significantly altered (Figure S6C,D). Morphological analysis of Iba1^+^ microglia further revealed no significant differences in soma area or branching complexity between the KA‐Veh and KA‐Bex groups during the chronic phase (Figure S6E–I). Together, these findings indicate that, in this cohort, therapeutic post‐SE administration of bexarotene was associated with lower seizure frequency and ectopic neuronal accumulation, lower astrocytic marker‐positive cell densities, and lower APOE^+^ astrocytic‐profile density in the hippocampal hilus, supporting further investigation of astrocyte‐associated lipid pathways as potential therapeutic targets in epilepsy.

### 2.7 Multi‐Omic Profiling and Molecular Validation Revealed Bexarotene‐Associated Metabolic and Signaling Changes after SE

To determine the effects of early post‐SE bexarotene intervention on hippocampal metabolic remodeling, mice received bexarotene or vehicle once daily for 7 consecutive days beginning 1 week after KA‐induced SE, and hippocampal tissues were collected at 2 weeks after SE (KA2W‐Bex and KA2W‐Veh groups). PLS‐DA analysis of metabolomic (Figure S7A) and lipidomic datasets (Figure S7B) obtained at 2 weeks post‐SE revealed separation between Bex‐ and Veh‐treated groups, indicating distinct overall metabolic and lipidomic profiles. Volcano plot analysis identified 169 metabolites with nominally altered abundance (56 increased and 113 decreased; Figure 7A). Of the 1,263 tested metabolites, 23 remained significant after BH correction (q < 0.05). After median scaling during lipidomic data preprocessing, 731 lipid species showed nominally altered abundance, including 390 increased and 341 decreased species (Figure 7C). Of 1,107 tested lipid species, 654 remained significant after BH correction (q < 0.05). KEGG over‐representation analysis was performed separately for increased and decreased features. Because only five increased metabolites had KEGG annotations, pathway analysis of this set was not considered sufficiently informative and was therefore not displayed in Figure 7B. Among decreased metabolites, efferocytosis, glycerophospholipid metabolism, and choline metabolism were enriched after BH correction (Figure 7B). In the lipidomic analysis, lipid species with increased abundance were enriched in polyunsaturated fatty acid metabolism and membrane phospholipid‐associated terms, whereas lipid species with decreased abundance were enriched in autophagy‐, sphingolipid‐, and lipid‐handling‐associated terms (Figure 7D). All pathway terms displayed in Figure 7D remained significant after BH correction. The top‐ranked lipid species showed bidirectional subclass‐specific changes: TG, PI, and PC species predominated among increased lipids, whereas Cer, PS, and a distinct subset of TG species predominated among decreased lipids (Figure 7E). Thus, bexarotene was associated with substantial remodeling of the hippocampal lipid profile rather than a uniform change across all lipid classes.

**FIGURE 7:**
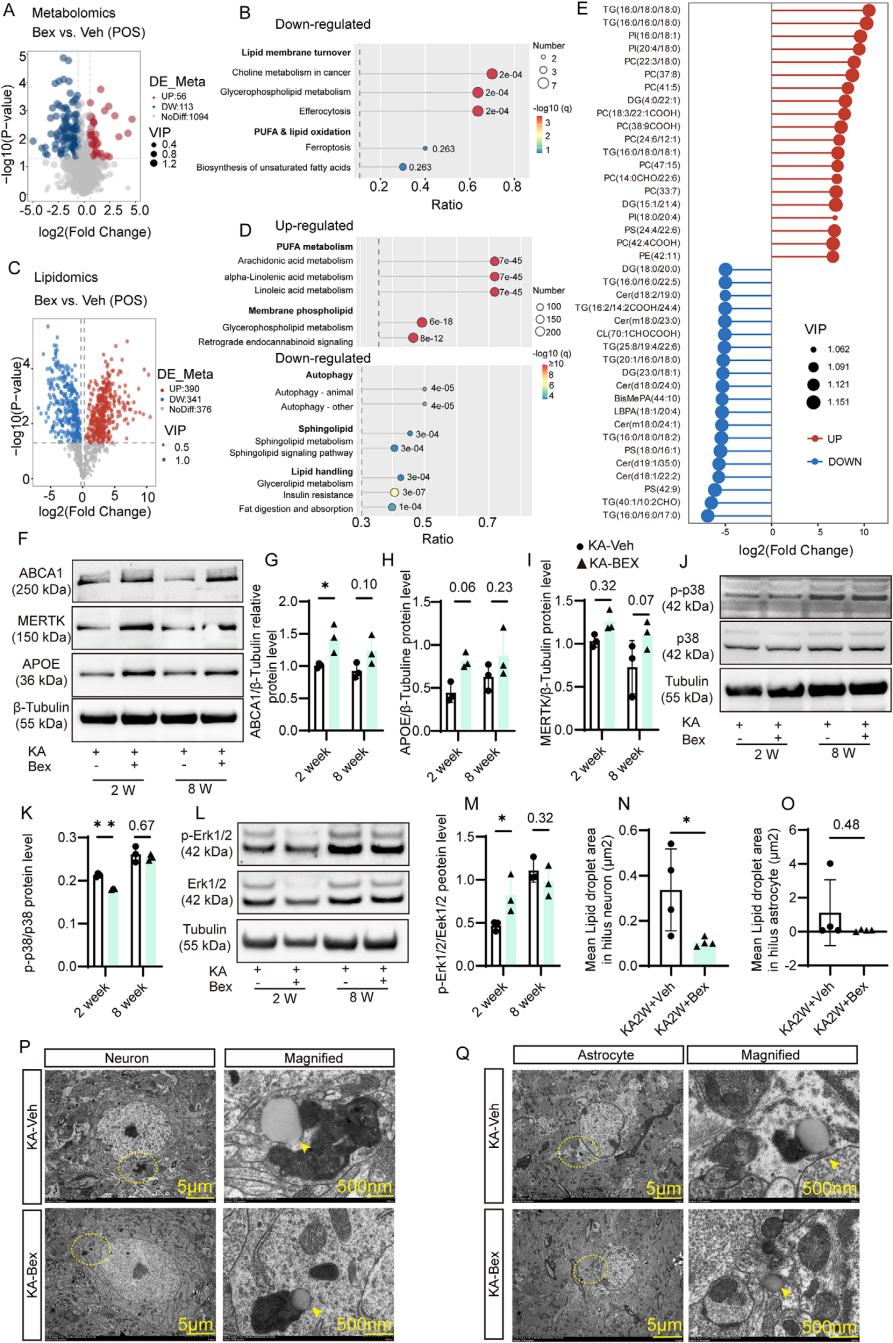
Multi‐omics profiling reveals bexarotene‐associated metabolic and lipidomic remodeling, alterations in lipid‐handling proteins and MAPK phosphorylation, and reduced neuronal lipid droplet area following KA‐induced SE. (A,C) Volcano plots showing differentially abundant metabolites (A) and lipid species (C) in hippocampal samples from KA2W‐Bex and KA2W‐Veh mice in POS. Red and blue dots indicate higher‐ and lower‐abundance features, respectively, in the Bex group relative to the Veh group; dot size indicates the VIP score. (B) KEGG over‐representation analysis of downregulated metabolites in KA2W‐Bex vs. KA2W‐Veh hippocampi. Upregulated and downregulated metabolites were analyzed separately; the upregulated set was not displayed because only five metabolites had KEGG annotations, providing too few annotated features for an informative pathway analysis. Dot size indicates the number of mapped metabolites. Color encodes −log_10_(*q*), where *q* is the Benjamini–Hochberg‐adjusted *p* value; *q* values are labeled beside the points. (D) KEGG over‐representation analyses of upregulated and downregulated lipid species in KA2W‐Bex vs. KA2W‐Veh hippocampi. Dot size indicates the number of mapped lipid species. Color encodes −log_10_(*q*), where *q* is the Benjamini–Hochberg‐adjusted *p* value; *q* values are labeled beside the points. (E) Lollipop plots showing the top 20 lipid species with the largest absolute log2 fold change in each direction. Red and blue indicate increased and decreased lipid species, respectively, in the Bex group; dot size represents the VIP score. (F–I) Western blot analysis (F) and quantification of ABCA1 (G), APOE (H) and MERTK protein levels normalized to β‐Tubulin in KA‐induced mice with/without Bex treatment at 2 and 8 weeks post‐SE. (J,K) Western blot analysis (J) and quantification of phosphorylated p38 (p‐p38) relative to total p38 protein levels (K), normalized to β‐Tubulin, in KA‐induced mice with or without Bex treatment at 2 and 8 weeks post‐SE. (L,M) Western blot analysis (L) and quantification of phosphorylated Erk1/2 (p‐Erk1/2) relative to total Erk1/2 protein levels (M), normalized to β‐Tubulin, in KA‐induced mice with or without Bex treatment at 2 and 8 weeks post‐SE. (N,O) Quantification of the mean total LD area per TEM image in neuronal (N) and astrocytic (O) within the hippocampal hilus of KA2W‐Veh and KA2W‐Bex groups. (P,Q) Representative TEM images of neurons (P) and astrocytes (Q) with corresponding magnified views. Yellow dashed circles indicate the regions shown at higher magnification, and yellow arrowheads indicate LDs. Scale bars: 5 µm (main panels), 500 nm (magnified insets). Data are presented as mean ± SD. Panels A‐E, G–I and K–M, *n = 3* mice per group. Panels N and O, *n = 4* mice per group. Protein‐expression data (G–I, K, and M) were analyzed by two‐way ANOVA followed by Sidak's multiple‐comparisons test. TEM data (N,O) were analyzed using the two‐sided Mann–Whitney *U* test. ^*^ *p* < 0.05 and ^**^ *p* < 0.01. Numbers above bars indicate exact non‐significant *P* values. For panels A–E and N–Q, data were obtained from the early post‐SE intervention cohort. Mice received Bex or matched Veh once daily for 7 consecutive days beginning 1 week after KA‐induced SE, and hippocampal tissues were collected at 2 weeks post‐SE. POS, positive‐ion mode; Veh, vehicle; Bex, bexarotene.

Because astrocyte‐mediated lipid transport through ABCA1 and APOE represents a central mechanism for cholesterol redistribution and debris handling in the injured brain, we next explored potential relationships between these proteins and lipid‐handling or phagocytic signaling components using computational analyses. PPI network analysis identified predicted associations connecting Abca1, Apoe, Mertk, and other lipid‐ and phagocytosis‐related proteins (Figure S7C). Molecular docking further generated a predicted ABCA1–APOE interaction pose with a docking score of −384.36 (Figure S7D). These computational results nominate potential molecular associations but do not establish direct binding or functional coordination among ABCA1, APOE, and MERTK.

To further evaluate temporal regulation of the proteins and signaling pathways implicated by the preceding analyses, we examined protein expression across two distinct pathological stages: an early subacute phase (bexarotene administered at 1 week and sampled at 2 weeks post‐SE) and a late chronic phase (bexarotene administered at 7 weeks and sampled at 8 weeks post‐SE). At 2 weeks post‐SE, Bex treatment significantly increased ABCA1 protein expression relative to Veh treatment (Figure 7F,G). Although APOE and MERTK protein levels showed higher mean values at this early time point, neither between‐group difference reached statistical significance after multiple‐comparisons correction (Cohen's d = 3.61 and 2.28, respectively; n = 3 per group; Figure 7F,H,I). These effect‐size estimates are descriptive and require confirmation in larger cohorts. In parallel, bexarotene treatment was associated with reduced p38 phosphorylation (Figure 7J,K; p < 0.01) and increased Erk1/2 phosphorylation (Figure 7L,M; p < 0.05). At 8 weeks post‐SE, no statistically significant Bex‐associated differences were detected for ABCA1, APOE, MERTK, p38 phosphorylation, or Erk1/2 phosphorylation. For MERTK at 8 weeks, the observed standardized difference was Cohen's d = 1.54 (p = 0.07); this estimate is descriptive and does not establish a treatment effect. Collectively, these findings show that the Bex‐associated changes in ABCA1 expression and MAPK phosphorylation were statistically significant at the early time point, whereas the corresponding late comparisons were not.

To assess LD area in the early post‐SE intervention cohort, TEM was performed on tissues collected at 2 weeks after SE (Figure 7N–Q). In hilar neurons, the mean LD area was 0.34 ± 0.18 µm^2^ in the KA2W+Veh group and 0.13 ± 0.04 µm^2^ in the KA2W+Bex group (n = 4 per group; Cohen's d = 1.58), with a statistically significant between‐group difference (Figure 7N,P). In hilar astrocytes, the corresponding values were 1.12 ± 1.94 and 0.07 ± 0.05 µm^2^, respectively (n = 4 per group; Cohen's d = 0.76) (Figure 7O,Q). This comparison was not statistically significant, and the KA2W+Veh group mean was strongly influenced by one verified mouse‐level value of 4.02 µm^2^, obtained from one eligible TEM image containing one identified LD; the other three mouse‐level values ranged from 0.07 to 0.30 µm^2^. In the GCL, mean neuronal LD area was 0.14 ± 0.06 µm^2^ in the KA2W+Veh group and 0.06 ± 0.05 µm^2^ in the KA2W+Bex group (n = 4 per group; Cohen's d = 1.55). In GCL astrocytes, the mean LD area was 0.01 ± 0.03 µm^2^ in the KA2W+Bex group compared with 0.11 ± 0.11 µm^2^ in the KA2W+Veh group (n = 4 per group; Cohen's d = 1.18) (Figure S7E–H). Neither GCL comparison reached statistical significance. Group means and standard deviations calculated from mouse‐level TEM measurements are provided in Table S4. Thus, the TEM data showed a statistically significant smaller LD area in hilar neurons after Bex treatment; the hilar astrocyte and GCL comparisons remain exploratory. Together with the multi‐omics and protein‐expression results, these findings support metabolic and lipid remodeling as candidate components of the early response to bexarotene treatment, while the functional roles of astrocyte‐associated lipid handling, ABCA1/APOE interactions, and MAPK signaling require further experimental validation.

## 3 Discussion

In the present study, seizure‐induced hippocampal remodeling was associated with exploratory lipidomic differences and larger mean mouse‐level lipid droplet area measures in the examined neuronal and astrocytic populations after SE. Immunofluorescence analyses further revealed PLIN2‐positive puncta near ectopic neuronal profiles and spatial association between ectopically located neurons and ABCA1‐positive cells, including GFAP‐positive astrocytic profiles, within the hippocampal hilus, suggesting a potential local association between astrocyte‐associated lipid handling and ectopic neuronal pathology during epileptogenesis. Multi‐omic profiling and molecular analyses further identified bexarotene‐associated changes in lipid‐related molecular profiles, ABCA1 expression, and MAPK phosphorylation during the early post‐SE period. Bexarotene treatment was associated with delayed acute seizure onset, reduced accumulation of ectopically located neurons, and lower chronic spontaneous seizure frequency, together with a smaller hilar neuronal LD area measure in the early treatment cohort and altered MAPK phosphorylation. Collectively, these findings support a model in which astrocyte‐associated lipid remodeling may be linked to structural and inflammatory changes during TLE development. However, the present data do not establish a direct ABCA1–APOE–MERTK signaling cascade or a causal role for altered lipid handling in these pathological processes.

Increasing evidence suggests that LDs function not only as metabolic storage organelles but also as dynamic regulators of innate immune signaling within the central nervous system [11]. Under pathological conditions, excessive lipid accumulation promotes oxidative stress, ferroptotic vulnerability [35], and transcriptional reprogramming of glial cells toward inflammatory phenotypes. In epilepsy, although previous lipidomic studies have identified widespread phospholipid remodeling following SE [8, 9], the cellular consequences of this lipid imbalance have remained incompletely understood. By integrating untargeted lipidomics with ultrastructural analysis, we observed nominal differences in hippocampal lipid profiles at 2 weeks after KA‐induced SE, accompanied by numerically higher mean LD area measures in neurons and astrocytes in both the hilus and GCL. No individual lipid candidate remained significant after multiple‐testing correction, and the mouse‐level TEM comparisons did not reach statistical significance. These observations are therefore exploratory and do not establish increased cellular lipid storage after SE. Given that neurons possess an inherently limited capacity for lipid storage and β‐oxidation, excess neuronal lipid accumulation may impose lipotoxic stress and could contribute to TLE progression [36, 37]. Furthermore, phospholipid remodeling may influence membrane stability and fluidity, which are important for neuronal polarization and migration [38, 39]. Thus, altered lipid homeostasis could potentially contribute to changes in cellular positioning and hippocampal structural remodeling during epileptogenesis. This interpretation is consistent with the aberrant cortical migration observed in specific lipid‐accumulated Mboat7 knockout models [38]. Together, these exploratory lipidomic and LD observations raise the question of whether altered lipid homeostasis is related to hippocampal structural remodeling and ectopic neuronal positioning during epileptogenesis. However, further studies are needed to determine whether these lipid changes directly contribute to these pathological alterations.

A key observation of this study was the spatial association of ectopically located hippocampal neurons with ABCA1‐positive cells, including GFAP‐positive astrocytic profiles, together with the presence of nearby PLIN2‐positive puncta. Although aberrant neurogenesis and hilar ectopic migration are well‐established contributors to epileptogenic circuit remodeling, their metabolic status has remained largely unexplored. Our results raise the possibility that ectopic neuronal populations are located within a lipid‐altered microenvironment to which neighboring astrocytes may respond. Consistent with emerging evidence that astrocytes participate in efferocytosis‐like clearance of neuronal debris through receptors such as MEGF10 and MERTK [40, 41], the spatial association observed here is compatible with, but does not establish, a role for astrocyte‐associated ABCA1 in the handling of neuron‐derived lipids. This interpretation is consistent with glial lipid‐handling programs observed in neurodegenerative disorders such as AD [42]. In our immunofluorescence analyses, a substantial proportion of ABCA1 immunoreactivity was associated with GFAP‐positive astrocytic profiles. Although ABCA1 signals showed limited apparent overlap with Iba1‐positive microglial profiles in the representative images, the relative cellular contributions were not quantitatively compared.

At 2 weeks after SE, bexarotene treatment was associated with broad metabolic and lipidomic remodeling, a significant increase in hippocampal ABCA1 protein expression, and a significantly smaller mean LD area per TEM image in hilar neurons. The corresponding comparisons in hilar astrocytes and the GCL did not reach statistical significance. Although APOE is a principal lipid carrier within the central nervous system and plays a central role in neuron‐to‐astrocyte lipid transfer under conditions of metabolic stress [43, 44], whole‐hippocampal APOE protein abundance did not differ significantly between groups at this time point (Figure 7H). MERTK protein abundance also did not differ significantly (Figure 7I). The APOE findings obtained under the two post‐SE treatment paradigms represent different outcomes. Following treatment during weeks 1–2 after SE, whole‐hippocampal APOE protein abundance was assessed at week 2 (Figure 7H). Following treatment initiated at week 2, the density of APOE‐positive astrocytic profiles in the hilus was lower at week 4 (Figure 6S). Hilar GFAP‐positive profile density was also lower in the latter cohort (Figure 6I,N), whereas the percentage of GFAP‐positive profiles labelled for APOE did not differ significantly (Figure 6T). Thus, the lower density of APOE‐positive profiles may partly accompany the lower density of GFAP‐positive profiles, but these measurements do not establish whether APOE expression changed on a per‐astrocyte basis. Differences in treatment and sampling times, anatomical scope, and endpoint prevent a direct comparison of the two APOE results. Previous studies have shown that MERTK signaling is associated with Erk1/2‐dependent regulation of apoptotic cell clearance and inflammatory resolution [45, 46]. These outcomes may also vary across post‐SE stages, although the present experiments cannot determine whether bexarotene has a stage‐dependent effect on astrocytic APOE expression.

MAPK pathways, including p38 and Erk1/2, have been implicated in inflammatory astrocyte responses, phagocytic regulation, and cell‐survival‐related processes in other contexts [47, 48, 49, 50]. In the present study, bexarotene treatment was associated with reduced p38 phosphorylation and increased Erk1/2 phosphorylation at 2 weeks post‐SE. However, these bulk hippocampal measurements do not establish that the observed changes in p38 and Erk1/2 phosphorylation mediate an astrocytic phenotypic transition. Thus, the early treatment‐associated changes observed here are consistent with a possible relationship between treatment‐associated lipid‐handling responses and MAPK signaling during epileptogenesis. However, the present data do not establish that ABCA1 directly regulates APOE, MERTK, p38, or Erk1/2 signaling, nor do they demonstrate enhanced efferocytosis. These mechanistic possibilities require validation using cell‐type‐specific and functional approaches.

The temporal analyses further suggest that the molecular response to bexarotene may vary across post‐SE stages. Bexarotene was associated with increased ABCA1 abundance and altered MAPK phosphorylation when administered during the earlier post‐SE period, whereas the corresponding comparisons did not reach statistical significance following treatment during the 7–8‐week interval. Notably, treatment initiated 14 days after SE was nevertheless associated with reduced spontaneous seizure frequency and lower densities of ectopically located neurons and astrocytic marker‐positive cells. These findings raise the possibility of stage‐dependent differences between molecular and phenotypic responses to bexarotene. However, the present experiments were not designed to define a discrete therapeutic window, and direct comparisons across treatment periods will require matched dosing, sampling, and outcome measures.

From a translational perspective, these results highlight astrocytic lipid metabolism as a potentially actionable target in TLE. However, several limitations should be considered. First, although bexarotene increased ABCA1 expression, it acts as a broad RXR agonist and may influence multiple downstream pathways; therefore, the observed effects cannot be attributed exclusively to ABCA1. Second, while spatial associations between ABCA1‐positive cells, including ABCA1/GFAP‐associated astrocytic profiles, and ectopically located neurons were observed, direct evidence of lipid transfer, engulfment, or intracellular lipid accumulation within these ectopic neurons was not obtained. Third, causal relationships between ABCA1‐associated responses, lipid transport, and reduced ectopic neuronal accumulation remain to be established, and will require cell‐type–specific genetic manipulation. Fourth, several endpoint‐specific analyses involved only three or four biological mice per group. Although the mouse was the experimental unit, these small cohorts limit the precision and reproducibility of the estimated differences. In particular, large Cohen's d values for nonsignificant comparisons are descriptive and do not establish treatment effects; conversely, statistically significant findings from small cohorts also require independent confirmation. The hilar astrocyte TEM mean in the early treatment cohort was particularly sensitive to one verified mouse‐level value, further limiting the robustness of that comparison. The chronic EEG findings were obtained from single cohorts with each bexarotene treatment paradigm; these paradigms differ in treatment timing and do not constitute independent replication of the same EEG result. The pilocarpine model was not used to replicate the chronic EEG outcomes. Larger, independently powered cohorts are needed to assess reproducibility. Fifth, continuous EEG was not recorded during acute KA‐induced SE in the pretreatment experiment. Although the behavioral seizure burden did not differ significantly among mice included in chronic EEG monitoring, bexarotene delayed the onset of severe behavioral seizures and one Bex‐pretreated mouse did not meet the predefined behavioral SE criterion. Therefore, the pretreatment experiment cannot fully distinguish an effect on epileptogenesis from a potential effect on acute KA susceptibility or electrographic SE severity. Sixth, only male mice were used in this study. The experimental design was not intended to assess sex‐dependent effects; therefore, the observed astrocyte‐associated lipid responses and bexarotene‐associated effects cannot be generalized to female mice. Future studies including both sexes are required to determine whether these responses differ by sex. Finally, the long‐term consequences of pharmacologically modulating astrocyte‐associated lipid responses on chronic network stability and seizure recurrence remain unclear.

## 4 Conclusions

In summary, our findings identify exploratory seizure‐associated differences in hippocampal lipid profiles and astrocyte‐associated ABCA1/APOE responses, together with spatial associations between ectopically located neuronal populations and ABCA1‐positive cells, including ABCA1/GFAP‐associated astrocytic profiles, in the hippocampal hilus. These observations support a potential relationship between local lipid‐associated remodeling, aberrant neuronal positioning, and reactive astrocytic responses during epileptogenesis. Bexarotene pretreatment and post‐SE administration were associated with lower chronic spontaneous seizure frequency and reduced densities of ectopically located neurons in their respective cohorts; pretreatment also delayed the onset of severe behavioral seizures. Across distinct treatment cohorts and time points, bexarotene was associated with changes in glial responses, lipid profiles, ABCA1 expression, and MAPK phosphorylation. Collectively, these findings support further investigation of astrocyte‐associated lipid pathways and their pharmacological modulation as potential therapeutic approaches for limiting epilepsy‐associated hippocampal remodeling.

## 5 Methods

### 5.1 Animals

Male C57BL/6J mice (8–10 weeks, 18–22 g) were purchased from Beijing Sibefu Bioscience Co., Ltd. (Beijing, China). All mice were housed in a specific pathogen‐free (SPF) environment with controlled temperature (22°C ± 2°C), humidity (50% ± 5%), and a 12‐h light/dark cycle (lights on at 7:00 AM). Standard laboratory chow and sterile water were available ad libitum. All animal experimental procedures were approved by the Animal Ethics Committee of Beijing Institute of Neurosurgery (Approval No. BNI202405004, Beijing, China) and conducted in strict accordance with the National Institutes of Health (NIH) Guide for the Care and Use of Laboratory Animals.

### 5.2 Experimental Design and Animal Model

#### 5.2.1 Kainic Acid (KA)‐Induced Status Epilepticus (SE) Mouse Model

To establish a KA‐induced SE model, male C57BL/6J mice received an initial intraperitoneal (i.p.) injection of KA (Abcam, USA, Cat# 120100) dissolved in sterile 0.9% saline at an initial dose of 25 mg/kg body weight. Mice were continuously monitored for behavioral seizure activity. Animals that did not develop a Racine stage IV or higher seizure during the initial observation period (60 min) received one supplemental KA dose (5 mg/kg, administered as a 1 mg/mL solution). No animal received more than one supplemental dose. These animals were then monitored for an additional 60 min. For each animal in which a Racine stage IV or higher seizure was first observed, this event was defined as the onset of a standardized 60‐min post‐onset monitoring period. Animals that failed to develop recurrent or sustained Racine stage ≥ IV behavioral seizures within this period, including animals exhibiting only a single isolated stage IV seizure, were classified as unsuccessful inductions and were excluded from downstream KA‐group analyses. During the standardized 60‐min monitoring period, recurrent or sustained Racine stage IV or higher seizures were recorded. Seizure activity was then terminated with diazepam (10 mg/kg, i.p.; Sigma–Aldrich, USA, Cat# D0899). Seizure severity was assessed according to the Racine scale. Animals that died during the monitoring period were recorded separately, and their outcomes were included in the animal‐disposition summary but were excluded from subsequent survival‐based endpoint analyses. Control (Con) mice received an equivalent volume of sterile 0.9% saline via i.p. injection.

#### 5.2.2 Pilocarpine‐Induced SE Mouse Model

To independently validate findings across distinct epileptogenic etiologies, a pilocarpine‐induced SE model was established. Mice were first administered scopolamine methyl bromide (1 mg/kg, i.p.; Sigma–Aldrich, USA, Cat# S8502) to reduce peripheral cholinergic effects. Thirty minutes later, SE was induced by a single intraperitoneal injection of pilocarpine hydrochloride (265 mg/kg; Abcam, UK, Cat# ab141301). Seizure severity was assessed using the Racine scale [51]. Consistent with the KA model, a standardized 60‐min monitoring period commenced immediately upon the first observation of a Racine stage IV or higher seizure. The identical successful‐induction criterion was applied: animals failing to develop recurrent or sustained Racine stage ≥ IV seizures (e.g., exhibiting only a single isolated seizure) were excluded. Seizure activity was terminated at 60 min post‐onset via diazepam administration (10 mg/kg, i.p.; Sigma–Aldrich, USA, Cat# D0899). Age‐matched control mice received identical treatments except that pilocarpine was replaced with an equivalent volume of sterile 0.9% saline. Following SE termination, mice received supportive care including subcutaneous saline and softened food.

For baseline characterization of both models, seizure burden was calculated for each mouse as the cumulative Racine score of all stage IV–VI seizures recorded during the standardized 60‐min post‐onset monitoring period. Detailed animal allocation, induction outcomes, exclusion due to failure to reach the predefined SE induction criterion, and mortality during induction and follow‐up for the KA‐ and pilocarpine‐induced SE cohorts are provided in Figure S1E–G, Figure S8, and Tables S1 and S2. Only surviving mice that met the predefined SE induction criteria were included in downstream analyses.

#### 5.2.3 Bexarotene Administration

Bexarotene (LGD1069, MCE, USA; cat. no. HY‐14171) was dissolved in a vehicle containing 5% DMSO, 40% PEG300, 5% Tween‐80, and 50% saline by sequential addition followed by sonication as previously described [52]. Bexarotene was administered by oral gavage at a dose of 100 mg/kg/day using four temporally defined treatment paradigms designed to evaluate stage‐dependent effects of RXR activation during epileptogenesis:

(i) Prophylactic pretreatment paradigm: Mice received bexarotene once daily for 7 consecutive days prior to KA administration to assess its effects on initial seizure susceptibility. Mortality and animal disposition for this cohort are summarized in Figures S5A, S8, and Table S1; the cohort size was not intended to evaluate treatment effects on survival. For subsequent chronic video‐EEG monitoring, electrodes were implanted 7 days after KA‐induced SE. Mice were allowed to recover for 7 days after electrode implantation, followed by continuous video‐EEG recording for 7 consecutive days (days 14–21 after SE).

(ii) Early post‐SE intervention paradigm: Mice received bexarotene once daily for 7 consecutive days beginning 1 week after KA‐induced SE. Brain tissues from this cohort were collected at the end of treatment for lipidomic and metabolomic analyses, transmission electron microscopy (TEM), and Western blotting (WB) to investigate early molecular and structural remodeling following SE.

(iii) Early chronic‐phase intervention paradigm: Mice received bexarotene once daily for 7 consecutive days starting 2 weeks after KA administration to evaluate its effects on spontaneous recurrent seizures (SRS) during the early chronic phase of epilepsy. For video‐EEG monitoring in this cohort, electrodes were implanted 14 days after KA‐induced SE. Bexarotene was administered during the subsequent 7‐day postoperative recovery period, followed by continuous video‐EEG recording for 7 consecutive days (days 21–28 after SE).

(iv) Late chronic‐phase intervention paradigm: Mice received bexarotene once daily for 7 consecutive days starting 7 weeks after KA administration. Brain tissues from this cohort were collected for WB analysis and compared with those from the early post‐SE intervention group to assess stage‐dependent responsiveness of the ABCA1–APOE signaling pathway.

Vehicle‐treated control animals received equivalent volumes of the matched solvent at corresponding time points.

A comprehensive experimental design and detailed animal allocation flowchart, including attrition and final sample sizes for each cohort, are provided in Figure S8.

### 5.3 Electroencephalogram (EEG) Recording

Mice were anesthetized with tribromoethanol (1.25% solution, i.p., 20 µL/g, Nanjing Aibei Biotechnology, Nanjing, China) and underwent stereotaxic implantation of EEG electrodes as previously described [53]. Six stainless steel screw electrodes were implanted epidurally over the skull. Recording electrodes were positioned symmetrically over the frontal and temporal cortices, while additional screws were placed over the occipital region. Among these, one occipital electrode served as the system ground (AGND), which is required to stabilize the signal and prevent charge accumulation in the animal, and another served as the reference electrode, consistent with the configuration of the Pinnacle headmount system. Continuous video‐EEG (vEEG) recordings were acquired for 7 consecutive days using a Sirenia acquisition system (version 2.1.0, Pinnacle Technology, Lawrence, KS, USA) at a sampling rate of 512 Hz. EEG data were analyzed using Sirenia software (version 1.7.5, Pinnacle Technology, Lawrence, KS, USA). Electrographic seizure‐like events (SLEs) were defined as high‐frequency rhythmic discharges > 3 Hz with amplitude ≥ 2 × baseline and duration ≥ 10 s [54]. SLEs were initially identified using automated detection in Sirenia and subsequently confirmed by manual visual inspection to ensure detection accuracy. The frequency, duration, and average power of SLEs were quantified for statistical analysis. For spectral analysis, EEG power was calculated using the built‐in power analysis function in Sirenia Seizure Pro software. According to the manufacturer's algorithm, power is defined as the root mean square (RMS) of the signal in the frequency domain:

$$Power=\sqrt{\sum_{n}\frac{{\left|x\left(f\right)\right|}^{2}}{n}}$$

where X(f) represents the Fourier‐transformed EEG signal and n is the number of data points. This metric reflects the overall signal energy within a specified frequency band. Average power values were computed automatically by the software over the duration of each detected event or within defined frequency ranges.

### 5.4 Sample Collection and Preparation

At designated time points (1, 2, 4, and 8 weeks) after SE, mice were deeply anesthetized with tribromoethanol (1.25% solution, i.p., 20 µL/g; Nanjing Aibei Biotechnology, Nanjing, China) and transcardially perfused with ice‐cold phosphate‐buffered saline (PBS, pH 7.4) followed by 4% paraformaldehyde (PFA). Brains were rapidly removed, post‐fixed in 4% PFA at 4°C for 24 h, and cryoprotected in a sucrose gradient (15% followed by 30% in PBS) at 4°C until fully submerged. Coronal hippocampal sections (40 µm) were prepared using a cryostat (Leica CM1950, Wetzlar, Germany) and stored in an antifreeze solution (30% glycerol and 30% ethylene glycol in PBS) at −20°C for subsequent immunofluorescence analysis.

For Western blotting and multi‐omics analyses, mice were euthanized by cervical dislocation at the indicated time points. Hippocampi were rapidly dissected on an ice‐cold platform, snap‐frozen in liquid nitrogen, and stored at −80°C until further processing to preserve protein and metabolite integrity.

### 5.5 Bioinformatics Analysis of Public Single‐Nucleus RNA Sequencing Datasets

To examine the cell‐type distribution of ABCA1/Abca1 and APOE/Apoe expression and to investigate astrocyte‐associated transcriptional changes in temporal lobe epilepsy (TLE), publicly available single‐nucleus RNA sequencing (snRNA‐seq) datasets were obtained from the Gene Expression Omnibus (GEO) database. For the human transcriptomic analysis, dataset GSE190453 was utilized. Initial data processing, quality control, and cell‐type annotation were performed according to the validated pipeline reported by Chen et al. [13]. Human cell‐type assignments were retained from the original dataset, in which astrocytes were identified using canonical markers including GFAP, FGFR3, GJA1, AQP4, and ALDH1L1. Although cell‐type annotations were retained from the original study metadata, differential‐expression and pathway‐enrichment analyses within the extracted astrocyte population were independently performed in the present study. For the murine analysis, the snRNA‐seq dataset GSE318570, generated from hippocampal tissues collected 4 days after KA or vehicle injection, was obtained. Raw 10× snRNA‐seq data were first processed using CellBender (v0.3.0) to remove ambient RNA contamination, and putative doublets were identified using the SOLO algorithm implemented in scvi‐tools (v1.4.0). Subsequent quality control filtering followed the criteria described in the original publication, including thresholds based on gene counts, UMI counts, and mitochondrial gene content [55]. Following quality control, the dataset was normalized using Scanpy (v1.11.4), and the top 3,000 highly variable genes were selected for downstream analysis. Dimensionality reduction and batch integration were performed using CONCORD (v1.0.12), followed by UMAP visualization and clustering. Unlike the human dataset, murine cell‐type annotation was performed independently in the present study based on canonical marker gene expression, with astrocytes identified using *Aldh1l1*, *Slc1a2*, and *Aqp4*. Cell‐type assignments were refined using CellAssign, and nuclei with inconsistent or ambiguous annotations were excluded. This procedure yielded a final dataset comprising 7,029 high‐quality nuclei across seven major cell types.

For the cell‐type expression analyses, normalized expression of ABCA1/Abca1 and APOE/Apoe was visualized across all annotated major cell populations in the human and murine datasets using violin plots. For downstream astrocyte‐focused analyses, astrocyte nuclei were extracted from each dataset. Differential gene expression analysis within the astrocyte population was performed using the Wilcoxon rank‐sum test implemented in Scanpy. To ensure cross‐species analytical consistency, candidate differentially expressed genes (DEGs) were defined using a uniform threshold of p < 0.05 and |log2(fold change)| > 0.25 (approximately corresponding to a fold change > 1.2). Functional interpretation of DEGs was performed using Gene Ontology (GO) Biological Process enrichment analysis implemented in g:Profiler (Python). All annotated genes in the corresponding dataset were used as the background reference. Enrichment *p* values were adjusted using the Benjamini–Hochberg procedure to control the false discovery rate, and terms with BH‐adjusted *p* < 0.05 were considered significantly enriched. For visualization, significantly enriched GO terms were selected using an identical predefined thematic procedure for the human and mouse datasets.

### 5.6 Multi‐Omics Analysis

#### 5.6.1 Untargeted Metabolomics Analysis

Hippocampal tissues (20 mg) were homogenized in 500 µL of pre‐cooled methanol/water (4:1, v/v) supplemented with L‐2‐chlorophenylalanine as an internal standard for metabolite extraction and normalization. The homogenate was sonicated on ice for 30 min and centrifuged at 12,000 × g for 15 min at 4°C. The supernatants were collected and filtered through 0.22 µm membranes prior to analysis. Untargeted metabolomic profiling was performed using a UHPLC‐MS/MS platform (Thermo Fisher Scientific) equipped with a Waters ACQUITY UPLC HSS T3 column (100 mm × 2.1 mm, 1.8 µm). The mobile phase comprised 0.1% formic acid in water (phase A) and 0.1% formic acid in acetonitrile (phase B). The gradient elution program was as follows: 0–1 min, 5% B; 1–9 min, 5%–95% B; 9–12 min, 95% B; 12–12.1 min, 95%–5% B; and 12.1–15 min, 5% B. The flow rate was maintained at 0.3 mL/min, and the column temperature was set to 40°C. MS/MS data were acquired in both positive and negative electrospray ionization modes (hereafter POS and NEG modes).

#### 5.6.2 Untargeted Lipidomics Analysis

For lipid extraction, hippocampal tissues (20 mg) were homogenized in 500 µL of pre‐cooled methyl tert‐butyl ether (MTBE)/methanol (3:1, v/v) supplemented with the isotope‐labeled internal standard mixture SPLASH Lipidomix Mass Spec Standard (Avanti Polar Lipids, USA). Samples were vortexed, sonicated on ice for 10 min, and incubated at room temperature for 1 h. Subsequently, 125 µL of water was added to induce phase separation, followed by centrifugation at 12,000 × g for 10 min at 4°C. The upper organic phase was collected, dried under nitrogen, reconstituted in 100 µL of methanol/isopropanol (1:1, v/v), and filtered through 0.22 µm membranes prior to analysis. Lipidomic profiling was performed using a Thermo Accucore C30 column (100 mm × 2.1 mm, 2.6 µm). The mobile phase consisted of acetonitrile/water (60:40, v/v; phase A) and isopropanol/acetonitrile (90:10, v/v; phase B), both supplemented with 10 mM ammonium formate and 0.1% formic acid (pH 3.2). The gradient program was as follows: 0–2 min, 40% B; 2–20 min, 40%–98% B; 20–24 min, 98% B; 24–25 min, 98%–40% B; and 25–30 min, 40% B. The flow rate was maintained at 0.3 mL/min with a column temperature of 55°C. MS/MS spectra were acquired in both POS and NEG modes.

#### 5.6.3 Multi‐Omics Data Processing and Bioinformatics Analysis

Raw metabolomic and lipidomic MS data were converted to the mzXML format using ProteoWizard (msconvert). For metabolomics, peak detection, alignment, and retention‐time correction were performed using XCMS. For lipidomic data, initial peak extraction and lipid identification were conducted using LipidSearch, followed by quantitative processing with XCMS. Metabolites and lipid species were annotated by matching accurate precursor *m/z* values and MS/MS fragmentation patterns against the KEGG, HMDB, and LIPID MAPS databases.

Both POS and NEG datasets were acquired and processed. Because the majority of confidently annotated differential metabolites and lipid species relevant to the present study were detected in POS mode, the multivariate analyses, differential‐feature visualizations, and pathway analyses presented in the main figures were based on the POS datasets. Multivariate statistical analyses were performed using MetaX. After log‐transformation and normalization, principal component analysis (PCA) and partial least squares‐discriminant analysis (PLS‐DA) were conducted to evaluate global metabolic alterations between experimental groups. PLS‐DA model performance was summarized using R2Y and Q2 values, and model overfitting was evaluated by permutation testing. Differential metabolites were identified using the combined criteria of variable importance in projection (VIP) ≥ 1.0, p < 0.05, and FC ≥ 1.5 or ≤ 0.667. Differential lipid species were identified using VIP ≥ 1.0, p < 0.05, and FC ≥ 1.2 or ≤ 0.833. For the Bex‐vs.‐Veh lipidomic comparison, a systematic between‐group intensity offset was identified during quality control; therefore, lipid intensities were additionally median‐scaled across samples before downstream differential and enrichment analyses. The differential‐feature thresholds were unchanged after median scaling.

Differential molecules were visualized using volcano plots and lollipop plots displaying log2 fold changes and VIP scores. Z‐score normalization and hierarchical clustering analysis (HCA) were performed to visualize expression patterns of differential lipids/metabolites. Finally, Kyoto Encyclopedia of Genes and Genomes (KEGG) over‐representation analysis was performed separately for increased and decreased metabolite or lipid sets using a one‐sided Fisher's exact test. The background consisted of all successfully annotated molecules detected in the corresponding dataset. P values were adjusted using the Benjamini–Hochberg procedure, and pathways with adjusted q < 0.05 were considered significantly enriched. Enrichment plots display the ratio of differential to background molecules within each pathway (x/y); point size represents the number of mapped molecules. For the metabolite analysis, the limited number of KEGG‐annotated increased metabolites precluded reliable pathway enrichment analysis for that direction.

### 5.7 Molecular Docking

The amino acid sequences for ABCA1 and APOE were obtained from the UniProt database (Accession numbers: P41233 for ABCA1 and P08226 for APOE). The three‐dimensional (3D) structures of both proteins were predicted using AlphaFold3. Subsequently, protein‐protein molecular docking was performed using the HDOCK server to evaluate potential interaction conformations between ABCA1 and APOE. Docking conformations were ranked according to the predicted docking score provided by the HDOCK scoring function, and the top‐ranked ABCA1‐APOE complex was selected for further structural analysis. To comprehensively characterize the predicted interaction mode, the binding interface and physicochemical parameters were evaluated using the online PDBePISA server. Finally, the 3D visualization of the interaction interface, including hydrogen bond and salt‐bridge mapping, was performed using PyMOL (v2.5).

### 5.8 Protein‐Protein Interaction (PPI) Network Construction

Core molecules (ABCA1, APOE, Mertk, etc.) were selected based on multi‐omics and molecular experimental results. The PPI network was constructed using the Search Tool for the Retrieval of Interacting Genes/Proteins (STRING, https://string‐db.org/) database with a confidence score ≥0.7.

### 5.9 Western Blot Analysis

Hippocampal tissues were lysed in RIPA buffer (Beyotime Biotechnology, Shanghai, China) supplemented with 1% phenylmethylsulfonyl fluoride (PMSF) and 1% phosphatase inhibitor cocktail (Beyotime) on ice for 30 min. Lysates were centrifuged at 12,000 ×g for 15 min at 4°C, and the supernatants were collected. Protein concentrations were determined using a BCA Protein Assay Kit (Thermo Fisher Scientific, Waltham, MA, USA). Equal amounts of protein (20 µg) were separated by 10% sodium dodecyl sulfate‐polyacrylamide gel electrophoresis (SDS‐PAGE) and transferred to polyvinylidene difluoride (PVDF) membranes (Millipore, Billerica, MA, USA). The membranes were blocked with 5% non‐fat milk in Tris‐buffered saline containing 0.1% Tween 20 (TBST) at room temperature for 1 h and incubated with primary antibodies overnight at 4°C. The primary antibodies used were as follows: anti‐ABCA1 (NB100‐2068, 1:1000, Novus Biologicals), anti‐APOE (83728‐4‐RR, 1:1000, Proteintech), anti‐MERTK (ab95925, 1:1000, Abcam), anti‐phospho‐p38 (28796‐1‐AP, 1:1000, Proteintech), anti‐p38 (66234‐1‐Ig, 1:1000, Proteintech), anti‐phospho‐Erk1/2 (28733‐1‐AP, 1:1000, Proteintech), anti‐Erk1/2 (11257‐1‐AP, 1:1000, Proteintech), anti‐GFAP (ab4674, 1:5000, Abcam), anti‐β‐tubulin (BF9210, 1:5000, Affinity Biosciences), and anti‐GAPDH (HY‐P80954A, 1:5000, MedChemExpress). After washing three times with TBST (10 min each), membranes were incubated with horseradish peroxidase (HRP)‐conjugated secondary antibodies (#611‐145‐122, 1:5000, Rockland) for 1 h at room temperature. Protein bands were visualized using an enhanced chemiluminescence (ECL) detection kit (Millipore) and imaged using a MINICHEMI Chemiluminescent Imaging System (Beijing Sage Creation Science Co., Ltd., Beijing, China).

### 5.10 Immunofluorescence

Coronal hippocampal sections (40 µm) were washed three times with PBS (10 min each) to remove the cryoprotectant solution. Sections were permeabilized with 0.3% Triton X‐100 in PBS for 30 min at room temperature, followed by blocking with 5% bovine serum albumin (BSA) in PBS for 1 h. Sections were then incubated overnight at 4°C with the following primary antibodies diluted in 5% BSA: mouse anti‐ABCA1 (NB100‐2068, 1:100, Novus Biologicals), rabbit anti‐Prox1 (ab199359, 1:200, Abcam), chicken anti‐GFAP (ab134436, 1:500, Abcam), rabbit anti‐doublecortin (4604, 1:200, Cell Signaling Technology), mouse anti‐PLIN2 (GP41, 1:200, Progen), rabbit anti‐APOE (83728‐4‐RR, 1:200, Proteintech), rabbit anti‐S100β (ab52642, 1:200, Abcam), mouse anti‐NeuN (ab104224, 1:200, Abcam), mouse anti‐MAP2 (67015‐1‐Ig, 1:200, Proteintech), and goat anti‐Iba1 (ab289874, 1:500, Abcam). After three PBS washes, sections were incubated with fluorophore‐conjugated secondary antibodies for 1 h at room temperature in the dark. The secondary antibodies included: donkey anti‐rabbit Alexa Fluor 594 (ab150064, 1:1000, Abcam), goat anti‐chicken Alexa Fluor 647 (ab150175, 1:1000, Abcam), donkey anti‐goat Alexa Fluor 647 (ab150135, 1:1000, Abcam), and donkey anti‐mouse Alexa Fluor 488 (ab150105, 1:1000, Abcam). Cell nuclei were subsequently counterstained with DAPI (SBA‐0100‐20, 1:1000, SouthernBiotech). Following three final washes with PBS, sections were mounted using an anti‐fade mounting medium (Beyotime Biotechnology). Fluorescence images were acquired using a confocal laser scanning microscope (Leica TCS SP8, Leica Microsystems, Wetzlar, Germany).

Confocal images were analyzed in Fiji (ImageJ) by an investigator blinded to the experimental groups. For in vivo analyses, one anatomically matched coronal hippocampal section and one predefined unilateral hilar ROI were analyzed per mouse. The hilus was defined as the area enclosed by the inner borders of the suprapyramidal and infrapyramidal blades, excluding the granule cell layer and subgranular zone. Images within each comparison were acquired and displayed using identical settings. For area‐based measurements, identical image‐processing and thresholding parameters were applied within each comparison. Marker‐positive cell bodies or morphologically identifiable cellular profiles were counted within the ROI and reported as cell density (cells/mm^2^), calculated as cell count divided by ROI area, or as the percentage of the relevant marker‐defined cell population, as specified in the figure legends. For direct double‐ or triple‐positive cell analyses, profiles were classified on a cell‐by‐cell basis only when the required marker signals were identified within the same morphologically identifiable cellular profile; merged‐image pixel overlap alone was not used to define double‐positive cells. Each data point represents one mouse.

For spatial‐apposition analyses, Prox1‐positive or DCX‐positive somata were segmented within the hilar ROI. A 1–3 µm annular region was generated around each soma. Neurons were classified as closely apposed to GFAP‐positive processes when GFAP signal covered ≥20% of this region; ABCA1‐associated apposition required ABCA1‐positive signal covering ≥5% of the same annulus; GFAP/ABCA1‐associated apposition additionally required both GFAP coverage of ≥20% and ABCA1‐positive signal covering ≥5% of the same annulus. Iba1‐associated apposition was defined as Iba1 signal covering ≥20% of the annular region. These measurements indicate close spatial apposition in two‐dimensional images and do not demonstrate physical contact or cellular co‐localization.

For in vitro analyses, identical thresholding parameters were applied within each comparison. APOE‐positive area in neurons was normalized to MAP2‐positive area. In astrocytes, APOE‐positive and ABCA1/APOE‐overlapping areas were normalized to GFAP‐positive area. For neuronal conditioned medium‐treated astrocytes, GFAP‐positive area fraction was calculated relative to total field area, while ABCA1/APOE/GFAP‐ and PLIN2/APOE/GFAP‐overlapping areas were normalized to GFAP‐positive area. Multiple fields were averaged for each independent culture preparation before statistical analysis. These area‐based measurements describe the extent of immunoreactivity and do not represent the number of positive cells or establish definitive cellular co‐localization; each data point represents one independent culture preparation.

### 5.11 Transmission Electron Microscopy (TEM)

Mice were anesthetized with tribromoethanol (1.25% solution, i.p., 20 µL/g; Nanjing Aibei Biotechnology, Nanjing, China) and transcardially perfused with 4% paraformaldehyde combined with 0.2% glutaraldehyde in 0.1 M PBS (pH 7.4). Hippocampal tissues were dissected and post‐fixed in 1% osmium tetroxide for 2 h, dehydrated through a graded ethanol series (30%–100%) followed by propylene oxide, and infiltrated with Durcupan resin (Electron Microscopy Sciences, USA) overnight at room temperature. Samples were embedded in resin blocks and polymerized at 55°C for 72 h. Ultrathin sections (65–80 nm) were prepared using a Leica Ultracut UC7 ultramicrotome, collected on copper grids, and stained with 2% uranyl acetate and lead citrate. Sections were examined using A Tecnai Spirit G2 TEM (FEI) operated at 80 kV. Predefined TEM fields containing identifiable neuronal or astrocytic profiles in the hippocampal hilus and GCL were acquired at 9300× magnification using an ORCA‐HR 10 MP camera, covering approximately 2000 µm^2^ of neuropil per image. Ultrastructural analyses were performed using AMT Image Capture Engine Software, to quantify LD accumulation in neurons and astrocytes.

For TEM quantification, all identifiable LDs within each predefined TEM image and within identifiable neuronal or astrocytic profiles were manually delineated as regions of interest (ROIs). For each image, the areas of all LD ROIs were summed to obtain total LD area per image (µm^2^). LD area was not normalized to total image area. Images without identifiable LDs were assigned a value of zero.

For each mouse, brain region, and cell type, all predefined TEM images acquired for each region and cell type were included. LD count per image was calculated as the total LD count divided by the number of analyzed images. Mean LD area was calculated as total LD area divided by LD count, and mean total LD area per image was calculated as total LD area divided by the number of analyzed images. For images without identifiable LDs, these measures were recorded as zero. Mouse‐level values were calculated by averaging image‐level measurements from the same mouse before statistical analysis. Thus, the mouse, rather than an individual cellular profile, TEM image, or LD, was the biological and statistical unit.

The Con/KA TEM cohort comprised three mice per group, whereas the KA2W‐Veh/KA2W‐Bex TEM cohort comprised four mice per group; all mice within each predefined TEM cohort were included in every region‐ and cell type‐specific analysis.

### 5.12 Primary Neuron Culture

Hippocampal tissues were isolated from embryonic day 17 (E17) mouse embryos on ice, minced into approximately 1mm^3^ fragments, and enzymatically digested with papain (2 mg/mL) at 37°C for 20 min. The tissue was gently triturated to obtain a single‐cell suspension and neutralized with Neurobasal medium (21103‐049, Gibco), supplemented with 2% B27 (17504‐044, Gibco), 1% penicillin‐streptomycin (SV30010, HyClone), and 1% GlutaMAX (35050‐061, Gibco). The suspension was filtered through a 40 µm cell strainer (431752, Corning). Cells were plated onto 6‐well plates pre‐coated with poly‐D‐lysine (20 µg/mL; P6407, Thermo Fisher Scientific) and maintained at 37°C in a humidified incubator with 5% CO_2_. Half of the culture medium was replaced every 3 days, and neurons were allowed to mature for 7 days in vitro before experimental treatments. Mature neurons were treated with KA (100 µM) for 12 h to generate neuronal conditioned medium (NCM). A subset of neurons was collected for analysis of APOE expression by immunofluorescence staining and qPCR.

### 5.13 Primary Astrocyte Culture

Primary astrocytes were isolated from the hippocampi of neonatal C57BL/6J mice. Hippocampal tissues were dissected on ice, minced into approximately 1 mm^3^ fragments in sterile PBS, and enzymatically digested with papain at 37°C for 20 min. Tissues were gently triturated to obtain a single‐cell suspension, and digestion was terminated with complete DMEM/F12 medium (11320‐033, Gibco) supplemented with 10% fetal bovine serum (FBS, 10099–141, Gibco) and 1% penicillin‐streptomycin. The suspension was filtered through a 40 µm strainer and centrifuged at 1500 × *g* for 5 min at 4°C. The cell pellet was resuspended in complete medium and seeded into culture flasks, which were maintained at 37°C in a humidified incubator with 5% CO_2_. The culture medium was replaced 24 h after plating and every 3 days thereafter. On day 6 in vitro, flasks were placed on an orbital shaker at 200 rpm for 16 h at 37°C to remove microglia. After removal of supernatant, fresh medium was added, and astrocytes were further cultured until approximately 90% confluence. Astrocytic identity and culture purity were simultaneously evaluated by immunofluorescence staining for GFAP and DAPI. Using ImageJ software, the percentage of GFAP‐positive cells relative to total DAPI‐positive cells was calculated. Quantification of 12 randomly selected fields (comprising 673 cells in total) demonstrated a culture purity of 86.9% ± 1.3% (mean ± SD), confirming a highly enriched astrocytic population. A subset of astrocytes was treated with KA (100 µM) to assess ABCA1 expression by immunofluorescence staining.

### 5.14 Astrocyte Culture With Neuronal Conditioned Medium

Primary neurons cultured for 7 days in vitro were treated with 100 µM KA for 12 h, and NCM was collected. Primary astrocytes were treated with bexarotene (50 nM) or vehicle for 12 h. Bexarotene was dissolved in DMSO and diluted in culture medium; vehicle‐treated cells received an equivalent volume of DMSO (final concentration, 0.1% v/v). Astrocytes were subsequently incubated with NCM for an additional 24 h to model neuron‐to‐astrocyte injury signaling during epileptogenic conditions. Following incubation, astrocytes were washed with PBS. Cells were then permeabilized with 0.3% PBST (PBS containing 0.3% Triton X‐100) for 30 min and subjected to immunofluorescence staining to assess expression of ABCA1, APOE, GFAP, and PLIN2, as well as PLIN2/APOE/GFAP overlapping immunoreactive areas in astrocytes.

### 5.15 RT‐PCR

Total RNA was extracted from primary hippocampal neurons and astrocytes using TRIzol reagent (Invitrogen, USA) according to the manufacturer's instructions. First‐strand cDNA was synthesized using a cDNA Synthesis Kit (Takara, Japan) following the manufacturer's protocol.

Quantitative real‐time PCR (qPCR) was performed using SYBR Green Master Mix (Thermo Fisher Scientific, USA) on a LightCycler 480 Real‐Time PCR System (Roche, Switzerland). The primer sequences used were as follows:

GAPDH: Forward 5′‐AGGTCGGTGTGAACGGATTTG‐3′ Reverse 5′‐TGTAGACCATGTAGTTGAGGTCA‐3′

Apoe: Forward 5′‐AGGGAAGATGGGGTTCTCTGG‐3′, Reverse 5′‐CGGCTAGGCATCCTGTCAGC‐3′

PCR cycling conditions were as follows: initial denaturation at 95°C for 3 min, followed by 40 cycles of 95°C for 10 s, 58°C for 20 s, and 72°C for 10 s. GAPDH served as the internal reference gene. Relative mRNA expression levels were calculated using the 2−ΔΔCT method and expressed as fold change relative to the control group.

### 5.16 Statistical Analysis

All data are presented as mean ± standard deviation (SD) with individual data points shown, unless otherwise indicated. Statistical analyses were performed using GraphPad Prism 9.0 (GraphPad Software, San Diego, CA, USA). For in vivo experiments, the individual mouse was considered the experimental unit; for in vitro experiments, an independent culture preparation was considered the experimental unit. Technical replicates, repeated measurements, microscopic fields, tissue sections, and individual cells obtained from the same mouse or independent culture preparation were aggregated or averaged to generate a single biological‐replicate value before statistical analysis. For two‐group comparisons of continuous outcomes, two‐sided unpaired Welch's t‐tests were used. For outcomes with clear non‐normal distributional characteristics, substantial zero inflation, ordinal properties, or count‐based measurements, two‐sided exact Mann–Whitney U tests were used. The choice of statistical test was based on the data type, distributional characteristics, and sample size, rather than solely on formal normality testing in small samples. For relative gene‐expression analyses evaluated against a prespecified hypothetical value of 1.0, two‐sided one‐sample t‐tests were used.

For analyses involving multiple groups and/or time points, ordinary two‐way analysis of variance (ANOVA) was performed where appropriate, followed by Sidak's multiple‐comparisons test for prespecified between‐group comparisons. All tests were two‐sided, and adjusted *p* values were used where multiple‐comparisons correction was applicable. *P* < 0.05 was considered statistically significant. Cohen's d was calculated for selected comparisons discussed in the Results to describe the observed standardized between‐group differences. Given the small biological cohorts, these estimates are imprecise and were not used to infer an effect from nonsignificant comparisons.

No observations were excluded on the basis of statistical outlier testing. Biological exclusions were limited to prespecified unsuccessful induction, mortality, or endpoint‐specific technical loss, as documented in Table S1.

## Author Contributions

L.C. conceptualized the study, designed and performed experiments, analyzed and curated the data, prepared the figures, and drafted the manuscript. X.‐R.S. contributed to methodology, validation, and data analysis. J.‐Y.C. contributed to software implementation, data curation, validation, and manuscript editing. Y.‐T.W., Y.N., and L.D. contributed to methodology, data acquisition, and formal analysis. J.‐M.C. contributed to visualization and manuscript editing. A.‐C.G. critically revised the manuscript. Q.Wu., J.‐P.W., and Q.Wa. supervised the study, acquired funding, and revised the manuscript. All authors reviewed and approved the final manuscript and agreed to be accountable for all aspects of the work.

## Funding

This study was supported by the National Natural Science Foundation of China (82501735, U24A20695, 82371449, 82471477, and 82160260), the National Key Research and Development Program of China (2022YFC2503800), the Beijing Natural Science Foundation (7232045 and Z200024), Capital's Funds for Health Improvement and Research (2024‐1‐2041), and the Yunnan Health Training Project of High‐Level Talents (D‐2024037).

## Ethics Statement

All animal experimental procedures were approved by the Animal Ethics Committee of Beijing Institute of Neurosurgery (Approval No. BNI202405004, Beijing, China) and were conducted in accordance with the National Institutes of Health Guide for the Care and Use of Laboratory Animals. No human participants or human specimens were involved in this study. The human single‐nucleus RNA‐sequencing dataset was obtained from the publicly available, de‐identified GEO dataset GSE190453, therefore, ethical approval and informed consent were the responsibility of the original study investigators.

## Consent

The authors have nothing to report.

## Conflicts of Interest

The authors declare no conflicts of interest.

## Supporting information




**Supporting File 1**: advs78329‐sup‐0001‐S1‐S8.docx.

**Supporting File 2**: advs78329‐sup‐0002‐Table1 Animal Allocation.docx.

**Supporting File 3**: advs78329‐sup‐0003‐Table2 Seizure Induction and Mortality.docx.

**Supporting File 4**: advs78329‐sup‐0004‐Table3.docx.

**Supporting File 5**: advs78329‐sup‐0005‐Table4.docx.

## Data Availability

Data supporting the findings of this study are provided in the Supporting Information or are available from the corresponding author upon reasonable request. Publicly available datasets analyzed in this study are available in the GEO repository under accession numbers GSE190453 and GSE318570.
