# Supplementary material for: Astrocyte‐Associated ABCA1/APOE Responses Accompany Hippocampal Lipid Remodeling and Ectopic Neuronal Accumulation During Epileptogenesis
Source: Adv Sci (Weinh). 2026 Oct 18:e78329. Online ahead of print. doi: 10.1002/advs.78329 (PMC13677000; doi:10.1002/advs.78329)
Supplement: Supplementary file 1 [file ADVS-9999-e78329-s001.docx]

Supplementary Table 1 Animal Allocation and Biological Replicate Numbers

| **Experimental cohort** | **Group or allocation stage** | **Initial animals in cohort, n** | **Chemoconvulsant-treated, n** | **Deaths during induction, n** | **Unsuccessful induction, n** | **Successful SE induction, n** | **Follow-up deaths, n** | **Final animals available, n** | **Endpoint allocation or disposition** |
| --- | --- | --- | --- | --- | --- | --- | --- | --- | --- |
| KA model | Control | 26 | N/A | N/A | N/A | N/A | N/A | 26 | Multi-omics, n=3; TEM, n=3; WB/IF: 1, 2, 4, and 8 weeks, n=5, 11, 5, and 5. |
| KA model | KA-SE | 33 | 33 | 2 | 2 | 29 | 3 | 26 | Multi-omics, n=3; TEM, n=3; WB/IF: 1, 2, 4, and 8 weeks, n=5, 11, 5, and 5. |
| Pilocarpine model | Control | 12 | N/A | N/A | N/A | N/A | N/A | 12 | WB/IF: 1, 2, 4, and 8 weeks, n=3 per time point. |
| Pilocarpine model | Pilocarpine-SE | 24 | 24 | 5 | 6 | 13 | 1 | 12 | WB/IF: 1, 2, 4, and 8 weeks, n=3 per time point. |
| 7-day Bex pretreatment | Veh, no KA (tissue collection) | 3 | N/A | N/A | N/A | N/A | N/A | 3 | Tissue collection, n=3. |
| 7-day Bex pretreatment | Bex, no KA (tissue collection) | 3 | N/A | N/A | N/A | N/A | N/A | 3 | Tissue collection, n=3. |
| 7-day Bex pretreatment plus KA | Veh + KA | 6 | 6 | 0 | 0 | 6 | 2 | 4 | Chronic video-EEG, n=4; WB/IF, n=4. |
| 7-day Bex pretreatment plus KA | Bex + KA | 6 | 6 | 0 | 1 | 5 | 1 | 4 | Chronic video-EEG, n=4; WB/IF, n=4. |
| **Therapeutic Bex administration** | **Total induction cohort** | **44** | **44** | **6** | **0** | **38** | **N/A** | **38** | **Successfully induced animals were allocated to the endpoint groups listed below.** |
| Therapeutic Bex administration | KA + Veh (post-SE allocation) | N/A | N/A | N/A | N/A | 6 | 2 | 4 | Chronic video-EEG, n=4; WB/IF, n=4. |
| Therapeutic Bex administration | KA + Bex (post-SE allocation) | N/A | N/A | N/A | N/A | 6 | 0 | 6 | Chronic video-EEG, n=4; WB/IF, n=4; tissue preserved, n=2. |
| Therapeutic Bex administration | KA2W + Veh (post-SE allocation) | N/A | N/A | N/A | N/A | 10 | 0 | 10 | Multi-omics, n=3; TEM, n=4; WB/IF, n=3. |
| Therapeutic Bex administration | KA2W + Bex (post-SE allocation) | N/A | N/A | N/A | N/A | 10 | 0 | 10 | Multi-omics, n=3; TEM, n=4; WB/IF, n=3. |
| Therapeutic Bex administration | KA8W + Veh (post-SE allocation) | N/A | N/A | N/A | N/A | 3 | 0 | 3 | WB/IF, n=3. |
| Therapeutic Bex administration | KA8W + Bex (post-SE allocation) | N/A | N/A | N/A | N/A | 3 | 0 | 3 | WB/IF, n=3. |

Note: Each mouse was treated as one biological replicate. For WB and IF analyses, left and right hippocampal hemispheres from the same mouse were assigned to the respective assays. “Deaths during induction” denotes deaths before successful SE induction; “follow-up deaths” denotes deaths after successful induction. N/A indicates that the field is not applicable or that allocation occurred after successful SE induction.
