# Supplementary material for: Astrocyte‐Associated ABCA1/APOE Responses Accompany Hippocampal Lipid Remodeling and Ectopic Neuronal Accumulation During Epileptogenesis
Source: Adv Sci (Weinh). 2026 Oct 18:e78329. Online ahead of print. doi: 10.1002/advs.78329 (PMC13677000; doi:10.1002/advs.78329)
Supplement: Supplementary file 2 [file ADVS-9999-e78329-s002.docx]

Supplementary Table 2 Seizure Induction Success and Mortality Across Chemoconvulsant Cohorts

| **Parameter** | **KA model** | **Pilocarpine model** | **Therapeutic KA cohort** |
| --- | --- | --- | --- |
| Total animals treated, n | 33 | 24 | 44 |
| Deaths during induction, n | 2 | 5 | 6 |
| **Acute induction mortality rate, %** | **6.06** | **20.83** | **13.64** |
| Unsuccessful inductions, n | 2 | 6 | 0 |
| Successful SE inductions, n | 29 | 13 | 38 |
| Follow-up deaths after successful SE induction, n | 3 | 1 | 2 |
| **Follow-up mortality rate among successfully induced animals, %** | **10.34** | **7.69** | **5.26** |
| Total deaths during induction and follow-up, n | 5 | 6 | 8 |
| **Overall mortality rate during induction and follow-up, %** | **15.15** | **25.00** | **18.18** |
| Final surviving animals after successful SE induction, n | 26 | 12 | 36 |
| Seizure frequency, median (IQR) | 4 (3, 6) | 2 (1, 3) | 3 (2, 3) |

Note: Acute induction mortality rate = deaths during induction / total animals treated. Follow-up mortality rate = follow-up deaths after successful SE induction / successful SE inductions. Overall mortality rate = total deaths during induction and follow-up / total animals treated. For the therapeutic KA cohort, the two follow-up deaths occurred in the KA + Veh endpoint-allocation group. Seizure-frequency statistics were calculated from animals with recorded Racine stage IV or higher seizure events. IQR = interquartile range (Q1–Q3).
