# Supplementary material for: Astrocyte‐Associated ABCA1/APOE Responses Accompany Hippocampal Lipid Remodeling and Ectopic Neuronal Accumulation During Epileptogenesis
Source: Adv Sci (Weinh). 2026 Oct 18:e78329. Online ahead of print. doi: 10.1002/advs.78329 (PMC13677000; doi:10.1002/advs.78329)
Supplement: Supplementary file 3 [file ADVS-9999-e78329-s003.docx]

**Supplementary Fig. S1 Single-nucleus transcriptomic analysis of hippocampal cell types and comparison of KA- and pilocarpine-induced SE models.**

(A) UMAP plot of major cell populations in the mouse hippocampal snRNA-seq dataset GSE318570. Nuclei are colored by annotated cell type.

(B) Dot plot of canonical marker-gene expression across annotated cell populations. Dot size indicates the proportion of nuclei expressing each gene, and color indicates mean expression level.

(C, D) Violin plots showing *ABCA1* expression in the human dataset (C) and *Abca1* expression in the mouse dataset (D) across major cell populations. *ABCA1/Abca1* expression was detected in astrocytes in both datasets.

(E) Experimental outcomes in KA- and pilocarpine-induced SE models, including total mice, deaths during induction, mice without qualifying seizures, survivors, and deaths during follow-up. Numbers above bars indicate mouse numbers.

(F, G) Number of qualifying Racine stage IV or higher seizures (F) and cumulative behavioral seizure burden (G) among mice with successful SE induction (KA, *n = 29*; pilocarpine, *n = 13*). Seizure burden was calculated as the cumulative Racine score of all qualifying seizures recorded during the standardized observation period.

For panels F and G, data are presented as mean ± SD and were assessed using the two-sided Mann–Whitney U test. ***P* < 0.01.

**Supplementary Fig. S2 *APOE/Apoe* expression across cell populations, astrocyte-associated APOE immunoreactivity, and hilar cellular changes following KA-SE.**

(A, B) Violin plots showing *APOE* expression in the human dataset (A) and *Apoe* expression in the mouse dataset (B) across major cell populations. *APOE/Apoe* expression was detected predominantly in astrocytes, with lower and heterogeneous expression in selected non-astrocytic populations.

(C) Representative images of APOE (red), GFAP (green), and DAPI (blue) in the mouse hippocampal hilus at 2 and 8 weeks after KA-induced SE. White dashed lines indicate hilar boundaries. White arrowheads in magnified insets indicate APOE immunoreactivity associated with GFAP⁺ astrocytic profiles. Scale bars: 20 μm (main panels) and 8 μm (magnified insets).

(D, E) Quantification of hilar GFAP⁺ cell density (D) and the proportion of APOE⁺/GFAP⁺ astrocytic profiles among total GFAP⁺ profiles (E) at 2 and 8 weeks after KA-induced SE (*n = 3* per group at each time point). Gray bars indicate control (Con), and red bars indicate SE.

(F) Representative immunofluorescence of NeuN (green), S100β (red), and DAPI (blue) in the dentate gyrus of control and SE mice at 1 week after SE. White dashed lines indicate the predefined hilar region of interest. Scale bars: 20 μm.

(G, H) Quantification of NeuN^+^ neuronal density (G) and S100β^+^ astrocyte density (H) within the hippocampal hilus at 1 week after SE (*n = 5* per group).

Data are presented as mean ± SD. Panels D and E were analyzed using two-way ANOVA followed by Sidak’s multiple-comparisons test. Panels G and H were analyzed using two-sided unpaired Welch’s t-tests. **P* < 0.05. Numbers above bars indicate exact non-significant *P* values.

**Supplementary Fig. S3 Temporal alterations in** PLIN2-positive puncta**, ABCA1-associated glial responses, and ectopic neuronal markers in the hippocampal hilus after KA-SE.**

(A, B) Representative immunofluorescence images showing PLIN2 (green) with Prox1 (red; A) or DCX (red; B) in control and SE mice at 1 week. DAPI is shown in blue in merged images. Yellow arrowheads highlight Prox1⁺ or DCX⁺ profiles spatially associated with PLIN2-positive puncta. Scale bars: 8 μm.

(C) Representative images of ABCA1 (green), GFAP (blue), Iba1 (magenta), and Prox1 (red) in the hippocampal hilus of control and SE mice at 2 and 4 weeks after SE. Boxed regions are shown at higher magnification, with corresponding single-channel images. Yellow arrowheads indicate representative Prox1⁺ cells adjacent to ABCA1/GFAP-associated astrocytic profiles. Scale bars: 20 μm (main panels), 8 μm (magnified insets).

(D–G) Quantification of hilar Prox1⁺ cell density (D), GFAP⁺ astrocyte density (E), Iba1⁺ microglial density (F), and the percentage of hilar Prox1⁺ cells adjacent to Iba1⁺ cells among total hilar Prox1⁺ cells (G) at 1, 2, and 4 weeks in control and SE mice (*n = 5* per group at each time point).

(H) Quantification of the percentage of hilar DCX⁺ profiles adjacent to Iba1⁺ cells among total hilar DCX⁺ profiles at 1, 2, 4, and 8 weeks in control and SE mice (*n = 5* per group at each time point).

(I) Representative images of DCX (red), ABCA1 (green), and Iba1 (magenta) in the hippocampal hilus of control and SE mice at 4 and 8 weeks after SE. Boxed regions are shown at higher magnification, with corresponding DCX and ABCA1 single-channel images. Yellow arrowheads indicate DCX⁺ profiles adjacent to ABCA1⁺ cells. Scale bars: 20 μm (main panels), 8 μm (magnified insets).

Data are presented as mean ± SD. Quantitative panels D–H were analyzed using two-way ANOVA followed by Sidak's multiple-comparisons test. **P* < 0.05, ***P* < 0.01, and ****P* < 0.001. Numbers above bars indicate exact non-significant *P* values.

**Supplementary Fig. S4. APOE-, ABCA1-, and** PLIN2-positive puncta **in primary neurons and astrocytes following KA exposure or neuronal conditioned medium.**

(A) Representative images of DAPI (blue), APOE (red), and MAP2 (green) in primary neurons from control and KA-treated cultures. White arrowheads indicate representative APOE immunoreactivity. Scale bar: 50 μm.

(B, C) Quantification of APOE^+^ immunoreactive area in primary neurons (B) and Apoe mRNA expression measured by RT–qPCR (C) in the Con and KA groups (B, *n = 6* independent cultures per group; C, *n = 3* independent cultures per group).

(D) Representative images of ABCA1 (green), APOE (red), and GFAP (grayscale) in control and KA-treated primary astrocytes. Enlarged merged and corresponding single-channel images are shown. Scale bars: 20 μm (main panels) and 7 μm (magnified insets).

(E, F) Quantification of APOE^+^ immunoreactive area (E) and ABCA1/APOE overlapping immunoreactive area (F) in primary astrocytes from the Con and KA groups (n = 6 per group).

(G, H) Quantification of GFAP^+^ area fraction (G) and ABCA1^+^/APOE^+^ area within GFAP^+^ astrocytes (H) following exposure to Veh-KA NCM or Bex-KA NCM. (*n = 6* independent culture preparations per group).

(I) Qualitative representative images of DAPI (blue), PLIN2-positive puncta (green), APOE (red), and GFAP (grayscale) in primary astrocytes exposed to Veh-KA NCM or Bex-KA NCM. Scale bar: 20 μm. PLIN2-positive puncta are shown for qualitative illustration; these images do not establish lipid-droplet abundance or definitive cellular co-localization.

(J) Quantification of PLIN2/APOE/GFAP-overlapping immunoreactive area normalized to GFAP^+^ area in primary astrocytes exposed to Veh-KA NCM or Bex-KA NCM (*n = 5* independent cultures per group).

Data are presented as mean ± SD. Panels B, E-H and J were analyzed using two-sided unpaired Welch’s t-tests. Panel C was analyzed using a two-sided one-sample t-test against a hypothetical value of 1.0.**P* < 0.05, ***P* < 0.01. Numbers above bars indicate exact non-significant *P* values.

**Supplementary Fig. S5. Effects of bexarotene pretreatment on acute seizure-related measures, and hilar cellular alterations after KA-SE.**

(A) Animal disposition in the 7-day Veh- and Bex-pretreatment cohorts following KA administration, including the total number of mice, deaths, animals without qualifying Racine stage IV or higher seizures, survivors, and animals included in chronic EEG recording.

(B, C) Number of Racine stage IV or higher seizures (B) and cumulative behavioral seizure burden (C) among Veh-KA and Bex-KA mice included in the EEG cohort (*n = 4* per group).

(D) Representative images of S100β (red), NeuN (green), Iba1 (cyan), and DAPI (blue) in Veh-KA and Bex-KA hippocampi. Dashed boxes in low-magnification images indicate regions shown at higher magnification; dashed lines delineate the predefined hilar region of interest. Scale bars: 200 μm (low-magnification images) and 20 μm (magnified images).

(E–G) Quantification of hilar S100β⁺ astrocyte density (E), NeuN⁺ neuronal density (F), and Iba1⁺ microglial density (G).

(H, I, K, L) Quantification of hilar Iba1⁺ microglial soma area (H), mean branch count (I), mean branch length (K), and mean maximal branch length (L).

(J) Representative Iba1 images, enlarged views, and corresponding skeleton reconstructions used for microglial morphometric analysis. Scale bar: 8 μm.

Data are presented as mean ± SD, with each dot representing one biological mouse (*n* = 4 per group). B and C were analyzed using the two-sided Mann–Whitney U test. E–L were analyzed using the two-sided unpaired Welch’s t-test. **P* < 0.05. Numbers above bars indicate exact non-significant *P* values.

**Supplementary Fig. S6. Effects of therapeutic bexarotene treatment on hilar neurons, astrocytes, and microglia at 4 weeks after KA-induced SE**

(A) Representative images of S100β (red), NeuN (green), Iba1 (magenta), and DAPI (blue) in KA-Veh and KA-Bex hippocampi. Dashed boxes in low-magnification images indicate regions shown at higher magnification; dashed lines delineate the predefined hilar region of interest. Scale bars: 200 μm (low-magnification images) and 20 μm (magnified images).

(B–D) Quantification of S100β^+^ astrocyte density (B), NeuN^+^ neuronal density (C), and Iba1^+^ microglial density (D) within the hippocampal hilus.

(E, F, H, I) Quantification of Iba1^+^ microglial soma area (E), mean branch count (F), mean branch length (H), and mean maximal branch length (I) within the hippocampal hilus.

(G) Representative Iba1 images, enlarged views, and corresponding skeleton reconstructions used for microglial morphometric analysis. Scale bar: 8 μm.

Data are presented as mean ± SD, with each dot representing one biological mouse (*n =4* per group). Quantitative panels B–F and H–I were analyzed using the two-sided unpaired Welch’s t-test. **P* < 0.05. Numbers above bars indicate exact non-significant *P* values.

**Supplementary Fig. S7. Bexarotene-associated metabolic profiles, predicted ABCA1/APOE interactions, and lipid droplet measurements in the granule cell layer (GCL) in KA-SE mice.**

(A, B) PLS-DA score plots of hippocampal metabolomic (A) and lipidomic (B) profiles in KA2W-Veh and KA2W-Bex mice (*n = 3* mice per group).

(C) Protein-protein interaction (PPI) network of core regulatory molecules, including Abca1, Apoe, Mertk, and associated signaling proteins.

(D) Predicted molecular docking model of ABCA1 (pink) and APOE (cyan). Key residues are shown as sticks, with H-bonds and hydrophobic interactions indicated by yellow and blue dashed lines, respectively. Docking score: −384.36.

(E, F) Representative TEM images of lipid droplets (LDs) in neuronal (E) and astrocytic (F) profiles within the hippocampal granule cell layer of KA2W-Veh and KA2W-Bex mice. Yellow dashed circles indicate regions shown at higher magnification, and yellow arrowheads indicate LDs. Scale bars: 5 μm (main panels) and 500 nm (magnified images).

(G, H) Quantification of the mean total LD area per TEM image in neuronal (G) and astrocytic (H) profiles within the hippocampal granule cell layer (*n = 4* mice per group).

Data are presented as mean ± SD. Panels G and H were analyzed using the two-sided Mann–Whitney U test. Exact *P* values are shown; neither comparison reached statistical significance. For panels A, B, and E–H, mice received Bex or matched Veh once daily for 7 consecutive days beginning 1 week after KA-induced SE, and hippocampi were collected at 2 weeks post-SE. POS, positive-ion mode; Veh, vehicle; Bex, bexarotene.

**Supplementary Fig. S8. Flowchart of experimental design, animal allocation, and attrition for *in vivo* and *in vitro* studies.**

This flowchart summarizes initial cohort sizes, animal allocation, and attrition across the KA-induced SE, pilocarpine-induced SE, bexarotene-pretreatment, and post-SE bexarotene-treatment experiments. Unsuccessful induction was defined as failure to develop qualifying Racine stage IV or higher behavioral seizures during the standardized observation period. Attrition included deaths during induction or follow-up. The flowchart indicates the final animal numbers allocated to each *in vivo* downstream analysis; independent culture sample sizes for in vitro experiments are reported in the corresponding figure legends. Con, control; SE, status epilepticus; KA, kainic acid; Veh, vehicle; Bex, bexarotene.
