# Supplementary material for: Astrocyte‐Associated ABCA1/APOE Responses Accompany Hippocampal Lipid Remodeling and Ectopic Neuronal Accumulation During Epileptogenesis
Source: Adv Sci (Weinh). 2026 Oct 18:e78329. Online ahead of print. doi: 10.1002/advs.78329 (PMC13677000; doi:10.1002/advs.78329)
Supplement: Supplementary file 4 [file ADVS-9999-e78329-s004.docx]

**Supplementary Table 3. Lipid droplet quantification in the hippocampal hilus and granule cell layer (GCL) of the dentate gyrus in control and KA-treated mice**

| **Brain region** | **Cell type** | **Parameter** | **Control (mean ± SD)** | **KA (mean ± SD)** | **Cohen’s d** |
| --- | --- | --- | --- | --- | --- |
| Hilus | Neuron | Mean LD count per TEM image | 0.00 ± 0.00 | 0.89 ± 0.19 | 6.62 |
| Hilus | Neuron | Mean area per LD (μm²) | 0.00 ± 0.00 | 0.59 ± 0.17 | 4.91 |
| Hilus | Neuron | Mean LD area per image (μm²) | 0.00 ± 0.00 | 0.52 ± 0.19 | 3.88 |
| Hilus | Astrocyte | Mean LD count per TEM image | 0.11 ± 0.19 | 0.67 ± 0.58 | 1.31 |
| Hilus | Astrocyte | Mean area per LD (μm²) | 0.03 ± 0.05 | 0.36 ± 0.45 | 1.04 |
| Hilus | Astrocyte | Mean LD area per image (μm²) | 0.01 ± 0.02 | 0.36 ± 0.44 | 1.14 |
| GCL | Neuron | Mean LD count per TEM image | 0.00 ± 0.00 | 1.00 ± 0.00 | Not estimable* |
| GCL | Neuron | Mean area per LD (μm²) | 0.00 ± 0.00 | 0.61 ± 0.46 | 1.84 |
| GCL | Neuron | Mean LD area per image (μm²) | 0.00 ± 0.00 | 0.61 ± 0.46 | 1.84 |
| GCL | Astrocyte | Mean LD count per TEM image | 0.00 ± 0.00 | 0.83 ± 0.29 | 2.86 |
| GCL | Astrocyte | Mean area per LD (μm²) | 0.00 ± 0.00 | 0.73 ± 0.98 | 1.30 |
| GCL | Astrocyte | Mean LD area per image (μm²) | 0.00 ± 0.00 | 0.42 ± 0.44 | 1.44 |

Note: For each mouse, mean LD count per TEM image, mean LD area per LD, and mean total LD area per TEM image were calculated from all available TEM images and cellular profiles and then summarized as mean ± SD. *n = 3* mice per group. Each data point represents one mouse; values from all available TEM images and cellular profiles from the same mouse were averaged before analysis. Images without identifiable LDs were assigned a value of zero. Cohen’s d was calculated using the pooled SD. *Not estimable because pooled within-group variance was zero. Effect sizes should be interpreted cautiously because several control or treatment groups contained very low or zero lipid-droplet counts, resulting in limited within-group variance and potentially unstable standardized effect-size estimates.
